# Capsaicin suppresses microglial inflammation through TRPV1–SSR4 interaction

**DOI:** 10.1186/s40035-026-00589-7

**Published:** 2026-10-21

**Authors:** Yifei Hou, Sichen Wang, Yuzhang Yang, Zhenzhen Zhao, Yuanyuan Xie, Xiaoyan Ouyang, Kaihua Zhang, Hongzhuan Chen, Jianrong Xu

**Affiliations:** 1 School of Integrative Medicine and State Key Laboratory of Discovery and Utilization of Functional Components in Traditional Chinese Medicine, Shanghai University of Traditional Chinese Medicine, Shanghai, China; 2 Shuguang Lab of Future Health, Shanghai Frontiers Science Center of TCM Chemical Biology, Shuguang Hospital, Shanghai University of Traditional Chinese Medicine, Shanghai, China; 3 State Key Laboratory of Brain Function and Disorders and MOE Frontiers Center for Brain Science, Center for Clinical Neuro-AI, Institute for Translational Brain Research, Fudan University, Shanghai, China; 4 Department of Emergency Medicine, Zhongshan Hospital, Fudan University, Shanghai, China

**Keywords:** Transient receptor potential vanilloid 1, Microglia, SSR4, Neuroinflammation, Interactome

## Abstract

**Background:**

Transient receptor potential vanilloid 1 (TRPV1) is increasingly recognized as an important regulator of microglial response during neuroinflammation, a key pathological feature of various neurodegenerative diseases such as Alzheimer’s disease. Capsaicin, the primary pungent component in chili peppers, executes its anti-inflammatory properties as a TRPV1 agonist. Although the mechanism of TRPV1 channel activation and signal transduction by capsaicin has been extensively studied, the protein–protein interaction-based mechanisms by which TRPV1 modulates microglial inflammatory responses in the context of neurodegeneration remains unclear.

**Methods:**

We used a lipopolysaccharide-induced acute neuroinflammation model to examine the effects of TRPV1 activation and pharmacological blockade of TRPV1 channel gating on microglial inflammatory responses. Proximity labeling-based proteomics, bioluminescence resonance energy transfer, and NanoLuc Binary Technology were used to identify and characterize TRPV1-interacting proteins. Signal sequence receptor subunit 4 (SSR4) knockdown and forced interaction between TRPV1 and SSR4 were performed to assess the functional contribution of the TRPV1–SSR4 interaction. Structural modeling, BRET-based domain mapping, and colocalization analyses were further used to examine the molecular and subcellular features of SSR4 recruitment by TRPV1.

**Results:**

In the neuroinflammatory model, the TRPV1 agonist capsaicin attenuated pro-inflammatory cytokine production, neuronal apoptosis, microgliosis, and microglial soma enlargement in a TRPV1-dependent manner. Notably, pharmacological blockade of TRPV1 channel gating by capsazepine did not abolish the anti-inflammatory action of capsaicin, suggesting an alternative mechanism of action beyond the canonical ligand-gated Ca^2^⁺ influx. Screening of TRPV1 prey proteins and validation strategy identified SSR4 as a capsaicin-responsive, TRPV1-associated protein. SSR4 knockdown in microglia significantly diminished the suppression of inflammatory responses caused by TRPV1 activation. Conversely, forced interaction between TRPV1 and SSR4 partially mimicked the anti-inflammatory effects of TRPV1 activation. Further structural and cellular analyses suggested that the transmembrane region of TRPV1 supports SSR4 recruitment.

**Conclusions:**

Our findings suggest that capsaicin suppresses microglial inflammation mediated by the TRPV1–SSR4 interaction, providing new insights into the chemical biology basis of TRPV1 interoceptive signaling and potential applications of natural TRPV1 agonists for modifying therapy of neuroinflammation-associated neurodegeneration.

**Supplementary Information:**

The online version contains supplementary material available at https://doi.org/10.1186/s40035-026-00589-7.

## Introduction

Neuroinflammation is a common pathological feature of many neurodegenerative disorders, including Alzheimer’s disease, Parkinson’s disease, and multiple sclerosis, and is also an important driver of disease progression [1, 2]. As resident immune cells of the central nervous system, microglia are central regulators of neuroinflammatory responses that contribute to both the pathogenesis and the progression of these neurodegenerative diseases [3, 4]. Defining molecular mechanisms that control the inflammatory activation of microglia is therefore important for understanding disease pathogenesis across neurodegenerative conditions and for developing more precise therapeutic strategies targeting neuroinflammation-driven neurodegeneration [5, 6].

Transient receptor potential vanilloid 1 (TRPV1) is a polymodal non-selective cation channel activated by noxious heat, extracellular protons, capsaicin, and other endogenous or exogenous ligands [7, 8]. Capsaicin, the primary pungent component responsible for the spiciness of chili peppers, is known as a classic TRPV1 agonist. Besides, TRPV1 serves as a common molecular target for bioactive constituents from acrid herbs in traditional Chinese medicines, such as evodiamine from *Evodia rutaecarpa*, thereby providing a pharmacological basis for studying how these natural compounds regulate sensory and inflammatory responses [9, 10]. Beyond its established role in sensory neurons, TRPV1 is also expressed in non-neuronal cells, including microglia, where it is implicated in immune and inflammatory regulation [11–13]. Although some studies have implicated TRPV1 in promoting microglial inflammation [13], findings from several neurodegenerative disease models support a protective role of TRPV1 activation, including improvements of microglial metabolism, autophagy, phagocytosis, and inflammatory status [11, 12, 14]. Nevertheless, current studies have largely reported attribution of TRPV1 function to its Ca^2^⁺ channel gating and downstream metabolic or autophagic effects. The molecular mechanisms by which TRPV1 modulates microglial inflammatory responses in the context of neurodegeneration therefore remain incompletely understood.

Emerging evidence suggests that TRPV1 may also regulate cellular signaling through mechanisms beyond ion permeation. TRPV1 has been reported to engage in protein–protein interactions, which influence its subcellular localization, stability, membrane trafficking, and downstream signaling output. For example, TRPV1 can interact with or form complexes with A-kinase anchoring proteins, GABA_A_ receptor-associated protein, the voltage-gated potassium channel β2 subunit, ankyrin repeat-rich membrane spanning/protein kinase D-interacting substrate, and the sigma-1 receptor [15–18]. Consistent with this concept, our previous study in a liver fibrosis model showed that TRPV1 suppresses hepatic stellate cell activation and inflammatory factor release by recruiting sterile alpha and TIR motif-containing 1 (SARM1), thereby slowing disease progression [19]. These findings raise the possibility that TRPV1 may regulate inflammation through context-dependent interactions with specific molecular partners.

Here, we asked whether TRPV1 controls microglial inflammatory responses through a protein-interaction-dependent mechanism. As the consequences of TRPV1 activation appear to vary across pathological contexts, we first defined the effect of TRPV1 activation in a microglial inflammatory model. Then pupylation-based interaction tagging (PUP-IT), bioluminescence resonance energy transfer (BRET), NanoLuc Binary Technology (NanoBiT), and co-immunoprecipitation were combined to identify TRPV1-associated proteins that mediate its immunoregulatory effects. Using this approach, we identified signal sequence receptor subunit 4 (SSR4), an endoplasmic reticulum (ER) translocon-associated protein [20, 21], as a capsaicin-responsive, TRPV1-associated protein and examined whether the TRPV1–SSR4 interaction contributes to the anti-inflammatory action of capsaicin in microglia.

## Materials and methods

### Drugs, reagents, antibodies, and experimental instruments

Detailed information of materials used in this study is provided in Tables S1–S3.

### Cell culture

BV2 mouse microglial cells were obtained from the Cell Bank of the Chinese Academy of Sciences (Shanghai, China). HMC3 human microglial cells (ATCC, CRL-3304), HEK293T cells (ATCC, CRL-3216), and CHO-K1 cells (ATCC, CCL-61) were obtained from ATCC. BV2 mouse microglial cells, HMC3 human microglial cells, HEK293T cells, and CHO-K1 cells were maintained under standard culture conditions. BV2 and HEK293T cells were cultured in Dulbecco’s modified Eagle’s medium (DMEM) supplemented with 10% fetal bovine serum (FBS) and 1% penicillin–streptomycin. HMC3 cells were cultured in Minimum Essential Medium (MEM) supplemented with 10% FBS and 1% penicillin–streptomycin. CHO-K1 cells were cultured in F12 medium supplemented with 10% FBS and 1% penicillin–streptomycin. All cells were maintained at 37 °C in a humidified incubator containing 5% CO_2_.

### Mice

Wild-type (WT) mice (6–8 weeks old, weighing 18–20 g) were purchased from Shanghai SLAC Laboratory Animal Co., Ltd. *Trpv1*-knockout (KO) mice were provided by the Shanghai Research Institute of Acupuncture and Meridian. All animals were maintained in a specific pathogen-free (SPF) facility at the Center for Animal Experimentation, Shanghai University of Traditional Chinese Medicine. Mouse genomic DNA for genotyping was extracted from tail tissues using the Quick Genotyping Assay Kit for Mouse Tail (Beyotime Biotechnology, Shanghai, China; D7283M) and amplified by standard PCR. Female mice were used throughout the study unless otherwise specified. Sample sizes are provided in the figures or figure legends for each experiment. Animals were randomly assigned to experimental groups using a random-number generator. Investigators were blinded to genotype and treatment during tissue processing, image acquisition, and quantitative analysis. Animals with injection failure, severe tissue damage, or poor staining quality, were excluded from subsequent data analysis.

### Isolation and culture of mouse primary microglia

Newborn mice (P0–P2) of the designated genotypes were immersed in 75% ethanol for 5 min. Brain tissues, excluding the cerebellum and olfactory bulb, were dissected, and the meninges were removed. The cortex and hippocampus were isolated under a dissection microscope and dissociated into a single-cell suspension by gentle pipetting, followed by centrifugation at 800 rpm for 3 min. After removal of the supernatant, 1 mL of trypsin solution was added and mixed for 30 s, followed by addition of 2 mL complete medium to neutralize the digestion. The suspension was passed through a 40-μm cell strainer and centrifuged at 1500× *g* for 5 min. The pellets were resuspended in DMEM/F12 containing 10% heat-inactivated FBS and 1% penicillin–streptomycin. Cells were seeded into 75 cm^2^ flasks. After 24 h, the medium was replaced, and from day 7 on, half of the medium was changed daily. On day 10, flasks were shaken at 900 rpm for 2 h at 37 °C to detach microglia, which were collected and cultured in DMEM with 10% FBS and 1% penicillin–streptomycin.

### Animal treatment

Lipopolysaccharide (LPS) was dissolved in sterile saline at 1 mg/mL. Capsaicin (HY-10448) and capsazepine (HY-15640) were purchased from MedChemExpress LLC (Monmouth Junction, NJ) and were prepared in a vehicle composed of 2% DMSO, 40% PEG300, 5% Tween-80, and 53% sterile saline. For both compounds, the solvents were added sequentially in the following order: DMSO, PEG300, Tween-80, and sterile saline. To induce an acute neuroinflammation model [22], 6-week-old female WT or *Trpv1* KO mice were randomly assigned to four groups: control, LPS, LPS + capsaicin, and LPS + capsaicin + capsazepine. Control mice received an intraperitoneal (i.p.) injection of 100 μL saline, followed 30 min later by a second equal-volume saline injection. Mice in the LPS group received i.p. saline injection, followed 30 min later by 2 mg/kg LPS. Mice in the LPS + capsaicin group received identical administrations to the LPS group, except an i.p. injection of 100 μL capsaicin (0.25 or 0.13 mg/kg) 30 min before LPS administration. In the LPS + capsaicin + capsazepine group, mice received i.p. injections of 100 μL capsaicin (0.25 or 0.13 mg/kg) and 100 μL capsazepine (5 mg/kg) 30 min before LPS administration. Brain tissues were collected 8 h after the final injection for subsequent experiments.

### Cell treatment

For in vitro experiments, LPS was dissolved in PBS at a stock concentration of 1 mg/mL. Capsaicin and capsazepine were dissolved in DMSO to prepare stock solutions at concentrations of 300 mM and 100 mM, respectively. All stock solutions were diluted to the indicated working concentrations immediately before use. BV2 microglial cells were retrieved from liquid nitrogen, placed on ice, and immediately thawed in a 37 °C water bath. After complete thawing, cells were transferred to a biosafety cabinet and suspended in 2 mL of complete medium within a 15-mL centrifuge tube. The suspension was centrifuged at 800 rpm for 3 min, the supernatant discarded, and the pellet resuspended in 1 mL of complete medium. Cells were then seeded into a T25 flask containing 5 mL of complete medium. When cultures reached 80%–90% confluence, cells were passaged and reseeded as required. After plating, cells were allowed to stabilize for 24 h. The medium was then replaced, and cells were treated with 2 μL of LPS (100 μg/mL), capsaicin (1 μM) and/or capsazepine (1 μM), mixed gently, and cultured at 37 °C with 5% CO_2_ for 24 h before subsequent experiments.

For immunofluorescence experiments, primary microglia were isolated and seeded onto glass confocal dishes. After 24 h of attachment, the cell density reached 30%–50%. After a complete medium change, the cells were treated with 1 μM capsaicin for 30 min, and then 1 μg/mL LPS was added and mixed thoroughly. The culture was continued at 37 °C in a 5% CO_2_ incubator for 24 h.

### siRNA knockdown

BV2 microglial cells were passaged and plated once they reached 70%–80% confluence. After 24 h of stabilization, the cells were transfected with siRNA to knock down *Trpv1* expression. All siRNAs used in this study were synthesized by Sangon Biotech (Shanghai) Co., Ltd. (Shanghai, China), and their sequences are provided in Table S2. The transfection solution was prepared using an RNAi transfection kit (D-nano Therapeutics Inc., Beijing, China; DN001-01). Briefly, 1 μL siRNA (20 μM) or negative control siRNA (NC) was sequentially mixed with 7 μL Reagent A and 2 μL Reagent B. The mixture was incubated at room temperature for 5 min, and then 190 μL Opti-MEM was added and mixed thoroughly. The solution was then added to the cell cultures in a 6-well plate. Drugs were administered to the cells 8 h after transfection.

### Enzyme-linked immunosorbent assay (ELISA)

Cytokine levels in cell culture supernatants or tissue homogenates were measured by ELISA. IL-6 and TNF-α concentrations were determined using ELISA kits (MultiScience, Hangzhou, China; EK206 and EK282) according to the manufacturer’s instructions. Briefly, samples and standards were added to pre-coated plates and incubated, followed by sequential incubation with detection antibodies and horseradish peroxidase (HRP)-conjugated secondary reagents. Color development was performed using the TMB substrate, and absorbance was measured at 450 nm using a microplate reader. No deviations from the manufacturer’s standard protocol were applied unless otherwise stated.

For inflammatory cytokine protein detection, mice were deeply anesthetized with sodium pentobarbital (40−60 mg/kg, intraperitoneally) and brain tissues obtained via perfusion with pre-chilled PBS. The tissues were rapidly frozen in liquid nitrogen and stored at − 80 °C for subsequent experiments. Frozen brain tissue was homogenized using a tissue grinder in a 1.5 mL centrifuge tube in the presence of protease and phosphatase inhibitors (APExBIO Technology LLC, Houston, TX; K1007/K1015). The homogenate was centrifuged at 5000 rpm for 10 min, and the supernatant was collected for ELISA. Samples were diluted 50-fold for IL-6 detection, but not for TNF-α detection.

### Quantitative RT-PCR (qRT-PCR)

Cell cultures were washed once with PBS, and RNA was extracted using an RNA extraction kit (Nanjing Vazyme Biotech Co., Ltd., Nanjing, China; RC112-01). Frozen mouse brain tissue was placed in a 1.5 mL centrifuge tube, and 1 mL RL lysis buffer was added. The mixture was homogenized using a tissue grinder, centrifuged at 5000 rpm for 10 min, and the supernatant was collected for subsequent qPCR analysis. RNA concentration was determined using a NanoDrop spectrophotometer (Thermo Fisher Scientific Inc., Waltham, MA; ND-ONE-W). Reverse transcription was performed in a 20 μL reaction system using a reverse transcription kit (Nanjing Vazyme Biotech Co., Ltd.; R323-01) to generate cDNA. The qPCR reaction mixture was prepared using a SYBR Green-based qPCR kit (Nanjing Vazyme Biotech Co., Ltd.; Q712-03) by mixing SYBR Green, primers, cDNA template, and nuclease-free water according to the kit instructions. qPCR was conducted in a QuantStudio™ 3 real-time qPCR instrument (Applied Biosystems, Foster City, CA; A28571). The qPCR program consisted of an initial denaturation at 95 °C for 30 s, followed by 40 cycles of denaturation at 95 °C for 10 s and annealing/extension at 60 °C for 30 s. Fluorescence signals were acquired at the end of each annealing/extension step. The 18S gene was used as the reference for normalizing qPCR data. The relative expression of TRPV1, IL-6, IL-1β, and TNF-α was calculated using the ΔΔCt method. Primer sequences are listed in Table S3.

### Co-immunoprecipitation (CO-IP)

CO-IP was performed to examine the interactions between TRPV1 and SSR4. HEK293T cells were seeded in 6-well plates at a density of 1×10^6^ cells per well and cultured in DMEM supplemented with 10% FBS at 37 °C in a humidified incubator containing 5% CO_2_. When cells reached approximately 70%–80% confluence, they were co-transfected with TRPV1-Flag and SSR4-mVenus-HA (both 1 μg) plasmids. Empty vectors were used as control.

After 24 h of transfection, cells were treated with capsaicin at 10 μM for 30 min. Control cells received the corresponding vehicle at the same final concentration. After treatment, cells were washed twice with ice-cold PBS and lysed on ice in ice-cold NP40 lysis buffer supplemented with protease and phosphatase inhibitor cocktails (APExBIO Technology LLC, Houston, TX; K1007/K1015). Cell lysates were incubated on ice for 30 min and then centrifuged at 12,000× *g* for 15 min at 4 °C to remove insoluble debris. The supernatants were collected, and protein concentrations were determined using a BCA protein assay. One-quarter of the protein lysate was used as the input sample and analyzed for Flag, biotin, or HA expression levels via Western blotting. The remaining three-quarters of the protein lysate was used for the magnetic bead pull-down assay. An appropriate volume of MonoRab™ Anti-DYKDDDDK magnetic beads (GenScript Biotech Corporation, Piscataway, NJ; L00835-1) was washed three times with the wash buffer prepared per the manufacturer’s instructions. The protein lysates were incubated with the magnetic beads at room temperature for 1 h. The sample was placed on a magnetic stand to allow bead adhesion to the tube wall. The supernatant was discarded, and the beads were thoroughly washed with wash buffer, re-adhered to the magnetic stand, and the wash buffer was discarded after each wash. This washing step was repeated three times. Subsequently, 60 μL of 1× loading buffer was added to the tube containing the magnetic beads, and the tube was heated in a metal bath at 100 °C for 5 min to elute the target protein from the beads. The tube was then placed on a magnetic stand to collect the beads. The supernatant contained the target protein, which was analyzed via Western blotting for Flag, biotin, or HA expression levels.

### Immunohistochemistry (IHC)

After transcardial perfusion with PBS followed by 4% paraformaldehyde (PFA), mouse brains were carefully dissected and post-fixed in 4% PFA at 4 °C overnight. The tissues were then dehydrated through graded ethanol solutions, cleared in xylene, and embedded in paraffin. Coronal brain sections were cut using a rotary microtome (Histological microtome, Leica Microsystems IR GmbH, Wetzlar, Germany, Leica RM2235) at a thickness of 4–5 μm, mounted onto glass slides, and stored at room temperature until further processing.

For IHC, the brain sections were dewaxed, rehydrated, and blocked with 5% goat serum to prevent non-specific binding. Tissue sections were incubated with a rabbit polyclonal anti-cleaved caspase-3 (Asp175) antibody (Cell Signaling Technology, Inc., Danvers, MA; 9661S), followed by a Multi-rAb® Polymer HRP-Goat Anti-Rabbit Recombinant Secondary Antibody (H+L) (Wuhan Sanying Biotechnology Co., Ltd. (Proteintech); Wuhan, China; RGAR011), stained with 3,3′-diaminobenzidine, and counterstained with hematoxylin. After dehydration and mounting, images were captured with an Axio Imager A2 microscope (Carl Zeiss Microscopy GmbH, Jena, Germany).

### Immunofluorescence (IF)

Primary microglia were grown on coverslips and treated as described. Cells were washed twice with pre-cooled PBS, fixed with 4% PFA for 15 min, and washed twice with PBS. Cells were permeabilized with 0.2% Triton X-100 for 30 min and washed twice with PBS. Cells were blocked with 1% BSA at room temperature for 1 h and washed twice with PBS. Cells were incubated overnight at 4 °C with anti-Iba1 (1:1000; FUJIFILM Wako Pure Chemical Corporation, Osaka, Japan; 019–19741) and/or anti-TRPV1 (1:100; Santa Cruz Biotechnology, Inc., Dallas, TX; sc-398417), diluted in 1% BSA, then washed twice with PBS. After two washes with PBS, the cells were incubated with goat anti-rabbit IgG (H+L) F(ab′)_2_ fragment conjugated to Alexa Fluor 594 (1:500; Cell Signaling Technology, Inc.; 8889S) and goat anti-mouse IgG (H+L) F(ab′)_2_ fragment conjugated to Alexa Fluor 488 (1:500; Cell Signaling Technology, Inc.; 4408S), diluted in 1% BSA. Cells were incubated at room temperature in the dark for 1 h, then washed twice with PBS. Then the cells were incubated with DAPI (1:10) for 30 min at room temperature in the dark, then washed twice with PBS. Finally, images were captured using an A1R MP+ multiphoton confocal microscope (Nikon Corporation, Tokyo, Japan).

For immunofluorescence on brain tissues, after transcardial perfusion with PBS followed by 4% PFA, brain tissues were dissected and post-fixed in 4% PFA at 4 °C overnight. The tissues were subsequently dehydrated through graded ethanol, cleared in xylene, and embedded in paraffin. Coronal brain sections were cut using a rotary microtome at a thickness of 4–5 μm. Sections were mounted onto glass slides and stored at room temperature until use. Following antigen retrieval, the brain tissue was permeabilized and blocked with 0.2% Triton X-100 and 1% BSA. Brain tissue sections were incubated with anti-TRPV1 (1:100; Santa Cruz Biotechnology, Inc.; sc-398417) or anti-Iba1 (1:1000; FUJIFILM Wako Pure Chemical Corporation, Osaka, Japan; 019–19741) antibody prepared in 1% BSA overnight at 4 °C, and then washed twice with PBS. The sections were mounted with ProLong™ Gold Antifade Mountant (Invitrogen, Thermo Fisher Scientific Inc., Waltham, MA; P36930). Finally, images were captured by two-photon microscopy.

For immunofluorescence colocalization analysis, HEK293T cells were seeded onto glass coverslips and transfected with the indicated plasmids, including TRPV1-Flag, SSR4-mVenus, and ER-targeted mCherry. For TRPV1 staining, cells were fixed with 4% PFA and stained with a CoraLite®594-conjugated DYKDDDDK Monoclonal antibody (Wuhan Sanying Biotechnology Co., Ltd. (Proteintech); CL594-66008). SSR4 and the ER were visualized by mVenus and ER-targeted mCherry fluorescence, respectively. Images were acquired using an A1R MP+ multiphoton confocal microscope (Nikon Corporation) under identical imaging settings for each comparison. Colocalization was assessed by merged fluorescence images and line-scan intensity analysis using ImageJ (National Institutes of Health, Bethesda, MD).

### Silver stain

Proteins were extracted from lysed cells and separated via SDS-PAGE. Per the manufacturer’s protocol (Beyotime, Shanghai, China; P0017S), the gel was processed through sequential steps: fixation, 30% ethanol wash, water wash, sensitization, water wash, silver staining, water wash, development, termination, and a final water wash. Silver-stained bands were visualized and imaged using an Amersham biomolecular imaging system (Cytiva, Marlborough, MA).

### Construction of plasmids

For PUP-IT plasmid construction, PafA was fused to the N- or C-terminus of TRPV1 with SGGGS linkers, producing TRPV1-PafA-Flag (C-terminal) and PafA-TRPV1-Flag (N-terminal) plasmids. Additionally, a substrate-specific BCCP-Pup(E) plasmid was designed for the ligase. A CAAX sequence was incorporated into the PafA system to generate the PafA-Flag-CAAX plasmid. The system included the pcDNA3.1 empty vector, TRPV1-Flag, PafA-Flag-CAAX (CAAX group), TRPV1-PafA-Flag (C group), PafA-TRPV1-Flag (N group), and BCCP-Pup(E) plasmids.

For TurboID Proximity Tagging System plasmid construction, TurboID was fused to the N- or C-terminus of TRPV1 with SGGGS linkers, producing TRPV1-TurboID-Flag (C-terminal) and TurboID-TRPV1-Flag (N-terminal) plasmids. The system included the pcDNA3.1 empty vector, TRPV1-Flag, TRPV1-TurboID-Flag (C-terminal), and TurboID-TRPV1-Flag (N-terminal) plasmids.

For NanoBiT System plasmid construction, the SmBiT subunit was fused to the C-terminus of TRPV1 to generate the TRPV1-SmBiT plasmid. The LgBiT subunit was fused to the C-terminus of SSR4, S100A9, HSP90AA1, and SERPINB12 to produce the SSR4-LgBiT, S100A9-LgBiT, HSP90AA1-LgBiT, and SERPINB12-LgBiT plasmids.

For BRET System plasmid construction, the Rluc8 donor was fused to the C-terminus of TRPV1 to generate the TRPV1-Rluc8 plasmid. The mVenus acceptor, tagged with HA, was fused to the C-terminus of SSR4, S100A9, and HSP90AA1 to produce the SSR4-mVenus-HA, S100A9-mVenus-HA, and HSP90AA1-mVenus-HA plasmids.

For chemically induced dimerization plasmid construction, FKBP12 was fused to the C-terminus of TRPV1 to generate the TRPV1-FKBP12 plasmid, and FRB was fused to the N-terminus of SSR4 to generate the FRB-SSR4 plasmid. Control plasmids expressing FKBP12 or FRB alone were used as negative controls. All constructs were synthesized and verified by Sanger sequencing by Sangon Biotech (Shanghai) Co., Ltd. (Shanghai, China) before use.

The detailed information is shown in Table S4.

### Proximity tagging system construction

For PUP-IT and TurboID proximity labeling, HEK293T cells were seeded at 1 × 10^6^ cells per well in 6-well plate and transfected with the indicated plasmids. The total amount of plasmid DNA was 2 μg per well, and plasmid ratios were kept constant among groups.

For PUP-IT construction [23, 24], the experimental groups included pcDNA3.1 control, TRPV1-Flag + BCCP-Pup(E) control, PafA-Flag-CAAX + BCCP-Pup(E) control, TRPV1-PafA-Flag (C-terminal) + BCCP-Pup(E) proximity labeling, and PafA-TRPV1-Flag (N-terminal) + BCCP-Pup(E) proximity labeling. After transfection with the respective plasmids, 4 μM biotin was added to activate the system. After 24 h, labeling was terminated by washing three times with ice-cold DPBS. Proteins were extracted, and biotinylated proteins were detected via CO-IP or immunofluorescence.

For TurboID proximity labeling system construction [25, 26], the experimental groups included pcDNA3.1 control, TRPV1-Flag control, TRPV1-TurboID-Flag (C-terminal) proximity labeling, and TurboID-TRPV1-Flag (N-terminal) proximity labeling. Twenty-four hours post-transfection, biotin (50 μM; MedChemExpress LLC; HY-B0511) was added to activate the system. After 10 min, labeling was terminated by washing three times with ice-cold DPBS. Biotinylated proteins were detected via CO-IP or immunofluorescence.

### Mass spectrometry identification experiment

Proteins from CO-IP were collected, subjected to reductive alkylation, digested overnight. Peptides were extracted, desalted, vacuum-dried, and resuspended in 0.1% formic acid. Then 5 μL of peptides was separated using an EASY-nLC 1200 system with an in-house-packed ReproSil-Pur 120 C18-AQ analytical column (1.9-μm particles, 75-μm inner diameter × 15 cm; Dr. Maisch GmbH, Ammerbuch-Entringen, Germany; material no. r119.aq.) at a flow rate of 300 nL/min. Mass spectrometry was conducted on a Q Exactive™ HF hybrid quadrupole-Orbitrap mass spectrometer (Thermo Fisher Scientific (Bremen) GmbH, Bremen, Germany) in data-dependent acquisition mode for tandem mass spectrometry. The full scan resolution was set to 60,000 (FWHM), with a mass-to-charge ratio range of m/z 350–1500. In the HCD fragmentation mode, the collision energy was set to 30%. Experimental data were submitted to the iProX platform for sharing. The mass spectrometry proteomics data were deposited to the ProteomeXchange Consortium (https://proteomecentral.proteomexchange.org) via the iProX partner repository with the dataset identifier PXD066698. Data were accessible via the provided link: https://proteomecentral.proteomexchange.org/cgi/GetDataset?ID=PXD066698 and iProX: https://www.iprox.cn/page/project.html?id=IPX0012747000.

### RNA-Seq analysis

Total RNA was extracted from BV2 microglia using the FastPure Cell/Tissue Total RNA Isolation Kit V2 (Vazyme Biotech Co., Ltd., Nanjing, Jiangsu, China; RC112-01) and assessed for quantity and quality with a NanoDrop 2000 UV–Vis spectrophotometer (Thermo Fisher Scientific Inc., Waltham, MA; ND-2000). The RNA was purified, fragmented, reverse transcribed into cDNA, and amplified. The libraries were assessed using an Agilent Bioanalyzer 2100, pooled, and sequenced on the NovaSeq 6000 Sequencing System (Illumina, Inc., San Diego, CA; 20012850). Sequencing quality was evaluated with FastQC, and low-quality reads were trimmed using Trimmomatic. Cleaned reads were mapped to the reference genome with HISAT2, and mapping quality was assessed using RSeQC and Qualimap. Gene expression levels were quantified using StringTie and normalized to transcripts per million. Differentially expressed genes (DEGs) were identified using DESeq2 with criteria of *q*-value ≤ 0.001 and |fold change|≥ 2, and expression differences were visualized using scatter plots and volcano plots. Gene Ontology (GO) and Kyoto Encyclopedia of Genes and Genomes (KEGG) pathway enrichment analyses were performed for DEGs with *q*-values < 0.05. Sequencing data generated from BV2 were submitted to the Sequence Read Archive with BioProject accession PRJNA1295138 and submission ID SUB15639787/SUB15644420. The data are publicly available, accessible via the following links: https://www.ncbi.nlm.nih.gov/sra/PRJNA1295138.

### Intracellular calcium mobilization assay

CHO-K1 cells were cultured and seeded into 96-well black polystyrene microtiter plates. At seeding, candidate siRNAs (CTBP2, SSR4, VDAC1, HSP90AA1, FKBP4, SERPINB12, S100A9, PTGES3) were added (Table S2). All siRNAs were custom synthesized by Sangon Biotech (Shanghai) Co., Ltd. (Shanghai, China). Twenty-four hours post-transfection, the TRPV1-Flag plasmid was transfected. After 24 h, calcium mobilization was assessed. An HBSS-based Fluo-4 AM loading solution was freshly prepared by supplementing 1× HBSS with 20 mM HEPES, 10 mM D-glucose, 2 mM probenecid (Sigma-Aldrich, Inc., St. Louis, MO; P8761), 0.05% Pluronic F-127 (Sigma-Aldrich, Inc.; P2443), and 2.5 μM Fluo-4 AM (Dojindo Laboratories Co., Ltd., Kumamoto, Japan; F311). The medium was discarded, cells were washed once with HBSS-based assay buffer (1× HBSS supplemented with 20 mM HEPES and 10 mM D-glucose), added with Fluo4-AM working solution, and incubated at 37 °C for 30 min. Cells were washed once with the assay buffer and maintained in 50 μL of the same buffer. The baseline calcium mobilization was measured using a PHERAstar® FSX multimode microplate reader (BMG LABTECH GmbH, Ortenberg, Germany). Subsequently, 50 μL of capsaicin working solution (gradient concentrations) was added, and the response was measured using the microplate reader.

### NanoBiT

The NanoBiT substrate furimazine working solution was prepared by adding 2.62 mL of anhydrous ethanol to a vial containing 1 mg of furimazine powder (ChemShuttle, Burlingame, CA; 185252). Then, 52.4 μL of 6 M hydrochloric acid was added. The solution was mixed thoroughly to yield a 1 mM furimazine stock solution. The stock solution was stored at − 20 °C or − 80 °C. The stock solution was diluted with deionized water to a 5 μM working solution. The working solution was prepared immediately before use. HEK-293 T cells were cultured and seeded into 96-well black polystyrene microtiter plates. Cells were transfected with the indicated expression plasmids at 60%–80% confluence using Hieff Trans™ Liposomal Transfection Reagent (Yeasen Biotechnology (Shanghai) Co., Ltd., Shanghai, China; 40802ES). The experiment included three groups: candidate interaction protein-LgBiT + HiBiT (positive control), candidate interaction protein-LgBiT + SmBiT (negative control), and candidate interaction protein-LgBiT + TRPV1-SmBiT (experimental). All plasmids were custom synthesized and verified by Sanger sequencing by Sangon Biotech (Shanghai) Co., Ltd. (Shanghai, China). Bioluminescence was measured after 24 h. The medium was removed, and cells were washed once with wash buffer. Then 90 μL of wash buffer was added, and 10 μL of furimazine substrate was added to achieve a 5 μM final concentration. The PHERAstar FSX microplate reader was activated with the NanoBiT-specific LUM plus filter set. The excitation wavelength was set to 480 nm, and bioluminescence intensity was measured in bottom-reading mode.

### BRET

The BRET substrate coelenterazine h working solution was prepared by adding 1.26 mL of anhydrous ethanol to a vial containing 500 μg of coelenterazine h powder (Yeasen Biotechnology (Shanghai) Co., Ltd., Shanghai, China; 40906ES10). Then, 24.4 μL of 6 M hydrochloric acid was added. The solution was mixed thoroughly to yield a 1 mM coelenterazine h stock solution. The stock solution was stored at − 20 °C or − 80 °C. The stock solution was diluted to a 5 μM working solution using a bench-top diluter. The working solution was prepared fresh for each use. HEK-293 T cells were cultured and seeded into 96-well black polystyrene microplates. Cells were transfected with the indicated expression plasmids at 60%–80% confluence. The experiment included two groups: mVenus + TRPV1-Rluc8 (negative control) and candidate interaction protein-mVenus + TRPV1-Rluc8 (experimental). The BRET assay was conducted 24 h after transfection. The medium was removed, and cells were washed once with wash buffer. Then 40 μL of wash buffer, 50 μL of TRPV1 modulator (gradient concentrations), and 10 μL of coelenterazine h substrate (5 μM final concentration) were added sequentially. The BRET1 filter set was configured, excitation was set at 480 nm, and fluorescence intensity was measured. The emission signal was recorded at 530 nm. The BRET ratio was calculated as follows: BRET ratio = [mVenus (535 ± 30 nm)/Rluc8 (475 ± 30 nm)] − [Rluc8 (535 nm)/Rluc8 (475 nm)] [27].

### Fluorescent microsphere phagocytosis assay

Primary microglia were isolated and cultured in 35-mm glass-bottom confocal dishes. Cells were allowed to adhere for 24 h and then transfected with SSR4 siRNA (Table S2). All siRNAs were custom synthesized by Sangon Biotech (Shanghai) Co., Ltd. (Shanghai, China). Fluorescent microspheres (1.0 μm mean particle diameter; 2.5% solids in aqueous suspension; Merck KGaA, Darmstadt, Germany; L2778) were incubated with FBS (100%, *v*/*v*) at room temperature in the dark for 30 min. The FBS containing microspheres was diluted to 10% in DMEM/F12 medium to prepare a complete medium. Twenty-four hours post-transfection, cells were incubated with fluorescent microspheres. 3 μL of the complete medium was added to each dish and incubated at room temperature in the dark for 30 min, followed by incubation at 37 °C in a 5% CO_2_ incubator for 3 h. Subsequently, immunofluorescence was performed.

### Rapamycin-induced dimerization

To artificially enhance the interaction between TRPV1 and SSR4, a chemically induced dimerization system was established based on the FKBP12/FRB pair [28]. Full-length TRPV1 was fused with FKBP12 to generate the TRPV1-FKBP12 construct, and SSR4 was fused with FRB to generate the FRB-SSR4 construct. The corresponding control vectors expressing FKBP12 or FRB alone were used as negative controls. HMC3 microglial cells were seeded in 6-well plates and transfected with the indicated plasmids. For each well, a total of 2 μg of the indicated plasmid DNA and 5 μL of Hieff Trans Liposomal Transfection Reagent (Yeasen Biotechnology (Shanghai) Co., Ltd., Shanghai, China; 40802ES) were separately diluted in 250 μL of serum-free Opti-MEM. The diluted transfection reagent was incubated at room temperature for 5 min, combined with the diluted plasmid DNA, and incubated for an additional 20 min to allow complex formation. The complexes were then added dropwise to the cells. After 24 h of transfection, cells were treated with LPS (1 μg/mL) in the presence or absence of rapamycin (200 nM, MedChemExpress LLC; HY-10219). Rapamycin was used to induce heterodimerization between FKBP12 and FRB, thereby forcing the proximity of TRPV1 and SSR4. Cells transfected with control constructs and treated with rapamycin were included to exclude nonspecific effects of rapamycin or the fusion tags. After treatment, total RNA was extracted using RNA extraction kit (Nanjing Vazyme Biotech Co., Ltd.; RC112-01), and reverse transcription was performed using cDNA synthesis kit (Nanjing Vazyme Biotech Co., Ltd.; R323-01). The mRNA level of TNF-α was determined by qRT-PCR using SYBR Green Master Mix (Nanjing Vazyme Biotech Co., Ltd.; Q712-03). Gene expression was normalized to GAPDH. This assay was designed to determine whether forced TRPV1–SSR4 association is sufficient to mimic the anti-inflammatory effect of capsaicin in microglia.

### Quantification and statistical analysis

Data are presented as mean ± SD or mean ± SEM, and were analyzed using GraphPad Prism 9.0. Paired comparisons were analyzed with *t*-tests, and multiple group comparisons were conducted using one-way ANOVA followed by Tukey’s multiple-comparisons test. Statistical significance was set at *P *< 0.05.

## Results

### Capsaicin attenuates the LPS-induced neuroinflammation by suppressing microglial activation in a TRPV1-dependent manner

**Fig. 1:**
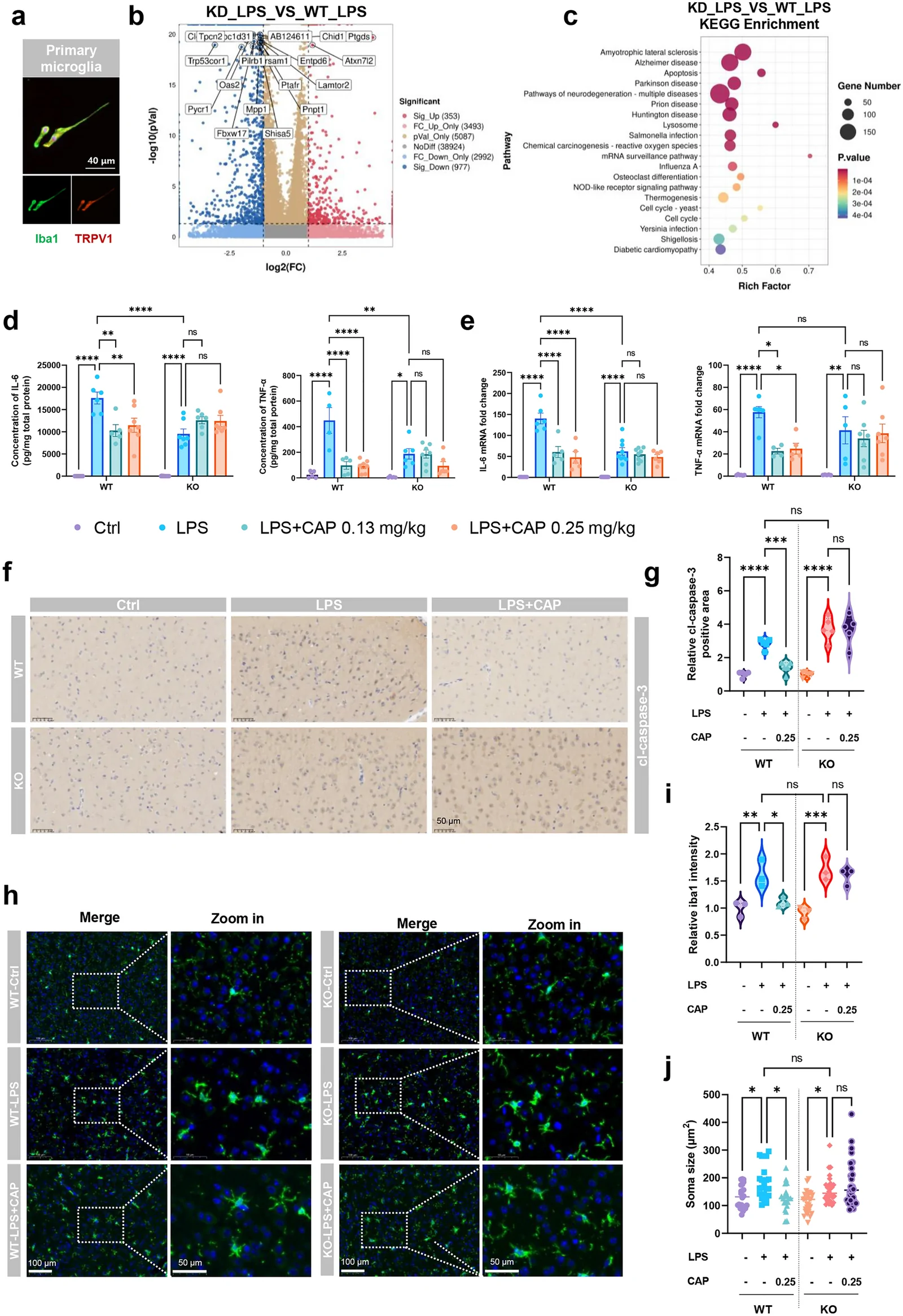
Capsaicin exerts anti-inflammatory effects via TRPV1 in a neuroinflammatory mouse model. **a** Fluorescence co-expression of TRPV1 (red) and Iba1 (green). Scale bar, 40 μm.** b** The effect of *Trpv1* knockdown (KD) on the transcriptome of BV2 under the inflammatory condition. **c** The effect of *Trpv1* KD on BV2 microglial pathways under the inflammatory condition.** d, e** Changes in protein and mRNA expression levels of IL-6 and TNF-α in the brain tissue of neuroinflammatory model mice after administration of capsaicin (CAP) in wild-type (WT) and *Trpv1* knockout (KO) mice. **f** Immunohistochemical staining of cleaved caspase-3 in brain tissues from WT and *Trpv1* KO mice in the Ctrl, LPS, and LPS + CAP groups. Scale bars, 50 μm. **g** Quantification of cl-caspase-3-positive areas from immunohistochemical staining (*n *= 3–5 mice per group). **h–j** Iba1 immunofluorescence staining of mouse brain tissue sections and statistical analysis of Iba1 immunofluorescence density in microglia (*n *= 3–5 mice per group) and microglial soma size (Each dot represents one individual cell. A total of *n *= 26–27 cells were analyzed from *n *= 3 independent biological replicates) in WT and *Trpv1* KO mice. Scale bars, 100 μm in overview images and 50 μm in enlarged images. Data represent mean ± SD. *P < 0.05, ** P < 0.01, *** P < 0.001, **** P < 0.0001, ns: not significant. *P* values were obtained with one-way ANOVA followed by Tukey’s multiple comparisons tests. CAP, capsaicin

We first confirmed the expression of TRPV1 in primary microglia isolated from C57BL/6 mice (Fig. 1a) and in brain sections from C57BL/6 mice (Fig. S1a, b). As the role of TRPV1 in neuroinflammation appears to vary across disease settings, we next sought to define its role in our LPS-induced inflammation model in vitro and in vivo. To this end, TRPV1 was knocked down in BV2 microglia (Fig. S1c–e). Transcriptomic analyses revealed that TRPV1 deficiency broadly altered the expression of inflammation-related genes and pathways under both control and LPS stimulation conditions (Fig. 1b, c; Figs. S2 and S3). However, TRPV1 deficiency had no significant effect on microglial morphology both in vitro (Fig. S4) and in vivo (Fig. S5).

We then asked whether activation of TRPV1 by capsaicin modulates neuroinflammation in vivo. In LPS-treated WT mice, capsaicin significantly reduced the mRNA expression and protein levels of IL-6 and TNF-α in brain tissue (Fig. 1d, e). Capsaicin also attenuated the LPS-induced neuronal apoptosis, as indicated by reduced cleaved caspase-3 staining (Fig. 1f, g; Fig. S6a). Importantly, these protective effects were lost in *Trpv1* KO mice (Fig. 1f, g; Fig. S6b), indicating that TRPV1 is required for the anti-neuroinflammatory effects of capsaicin. Consistently, in WT mice, LPS markedly increased the relative Iba1-positive area and enlarged the microglial soma area, while these changes were reversed by capsaicin (Figs. 1h–j, S7a). However, these effects of capsaicin were not observed in *Trpv1* KO mice (Fig. 1h–j, Fig. S7b), although LPS still induced microglial activation in the absence of TRPV1. Together, these results show that capsaicin attenuates the LPS-induced neuroinflammation in a TRPV1-dependent manner.

### Pharmacological blockade of TRPV1 channel gating fails to reverse the anti-inflammatory effect of capsaicin in microglia

**Fig. 2:**
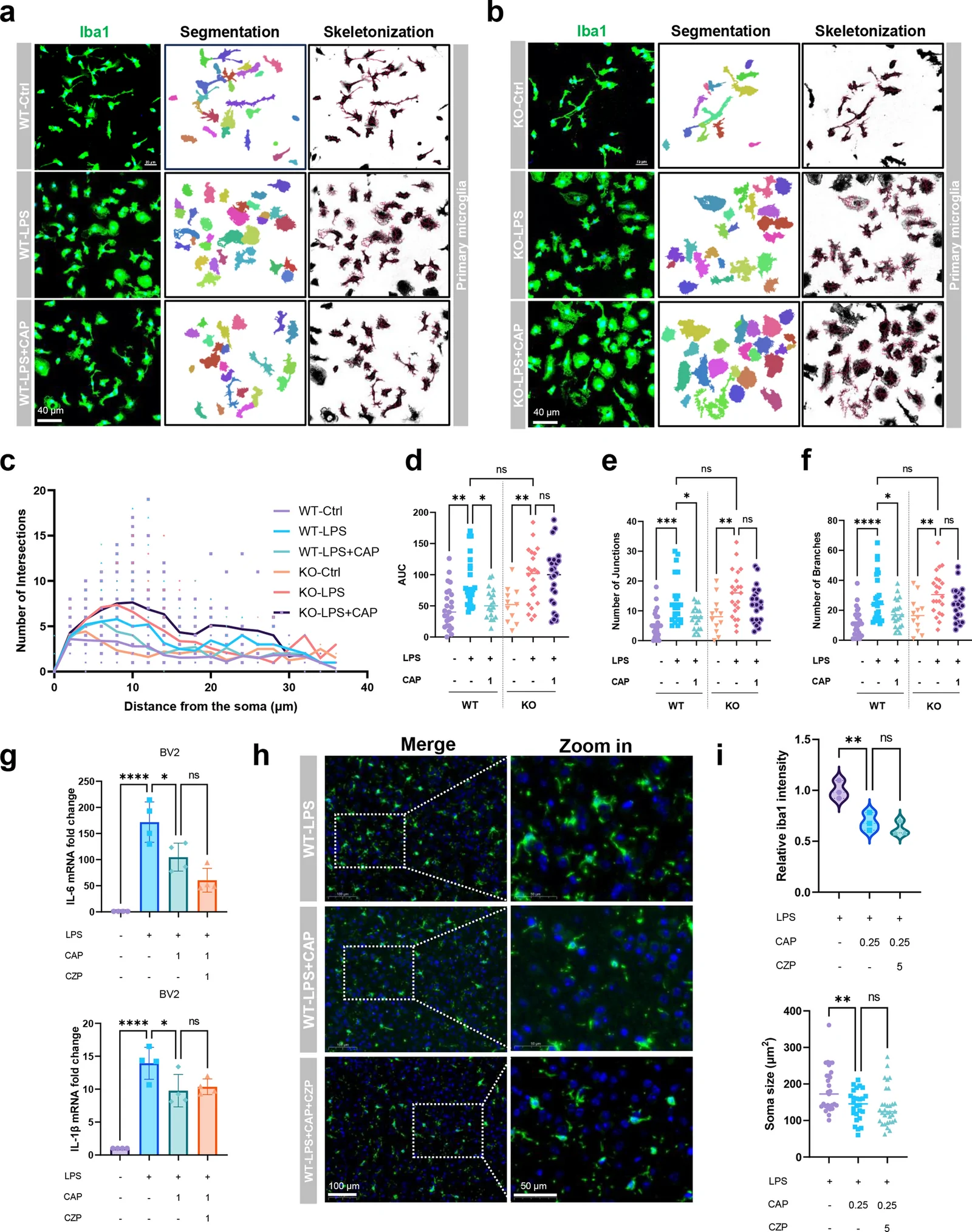
Blocking the channel function of TRPV1 cannot reverse the anti-neuroinflammatory effects of capsaicin in microglia. **a, b** Iba1 immunofluorescence staining, segmentation and skeletonization analysis of primary microglia from WT and *Trpv1* KO mice. Scale bars, 40 μm. **c–f** Analysis of microglial skeletonization and Sholl analysis, including cellular complexity (**c**), the area under the curve calculation (**d**), the number of junctions (**e**) and the number of branches (**f**) (Each dot represents one individual cell. A total of *n* = 12–27 cells were analyzed from *n* = 3 independent biological replicates). **g** Effects of capsaicin and capsazepine on IL-6 levels and IL-1β levels in BV2 microglia in vitro (*n* = 4 independent biological replicates). **h, i** Effects of capsaicin and capsazepine on microglial inflammatory responses in vivo, including quantification of microglial fluorescence intensity (*n* = 4 independent biological replicates) and soma size (Each dot represents one individual cell. A total of *n* = 26–32 cells were analyzed from *n* = 3 independent biological replicates). Data represent mean ± SD. *P < 0.05, **P < 0.01, ***P < 0.001, ****P < 0.0001, ns: not significant. *P* values were obtained with one-way ANOVA followed by Tukey’s multiple comparisons tests. CAP, capsaicin; CZP, capsazepine

We next examined whether capsaicin also modulates microglial morphology. Primary microglia from WT and *Trpv1* KO mice were stimulated with LPS (1 μg/mL) and treated with capsaicin (1 μM), followed by immunofluorescence staining, skeleton analysis, and Sholl analysis (Fig. 2a–f). Under control conditions, microglia displayed a ramified morphology with elongated processes. LPS induced a typical activated phenotype characterized by enlarged cell bodies, shortened processes, and an amoeboid appearance [29]. Quantitative skeleton analysis showed that LPS increased the number of branches and junctions. Sholl analysis further revealed a denser intersection profile, consistent with a compact activated morphology. Capsaicin partially reversed these changes toward the resting state, including branch number, junction number, area under the curve, and overall Sholl profile in WT primary microglia (Fig. 2a, c–f). In contrast, this morphological rescue was markedly weakened in TRPV1-deficient microglia (Fig. 2b–f), indicating that capsaicin suppresses microglial activation morphology largely through TRPV1.

TRPV1 is a classical non-selective cation channel that mediates Ca^2^⁺ influx upon agonist stimulation [30]. We therefore asked whether the anti-inflammatory effect of capsaicin depends on this canonical channel gating. To test this, we introduced the TRPV1 antagonist capsazepine [31] in both in vitro and in vivo models, to pharmacologically inhibit the capsaicin-evoked TRPV1 channel gating. In BV2 microglia, capsaicin significantly reduced IL-6 and IL-1β expression, but co-treatment with capsazepine at 1 μM failed to reverse this effect (Fig. 2g). Likewise, in the LPS-treated WT mice, capsazepine did not abolish the effects of capsaicin in reducing microgliosis or soma enlargement (Fig. 2h, i; Fig. S8a). To further control for TRPV1-independent pharmacological effects of capsazepine, we examined the same treatment conditions in BV2 microglia after *Trpv1* siRNA knockdown. In control cells, capsaicin suppressed the LPS-induced IL-6 expression, whereas capsazepine did not reverse the effect of capsaicin. After *Trpv1* knockdown, capsaicin, capsazepine, or their combination failed to show any effects on the LPS-induced IL-6 expression, supporting a role for TRPV1 in mediating the effects of capsaicin (Fig. S8b). These results suggest the involvement of an alternative, noncanonical mechanism of TRPV1.

### Engineered proximity labeling and interaction screening identify SSR4 as a candidate TRPV1-interacting protein

**Fig. 3:**
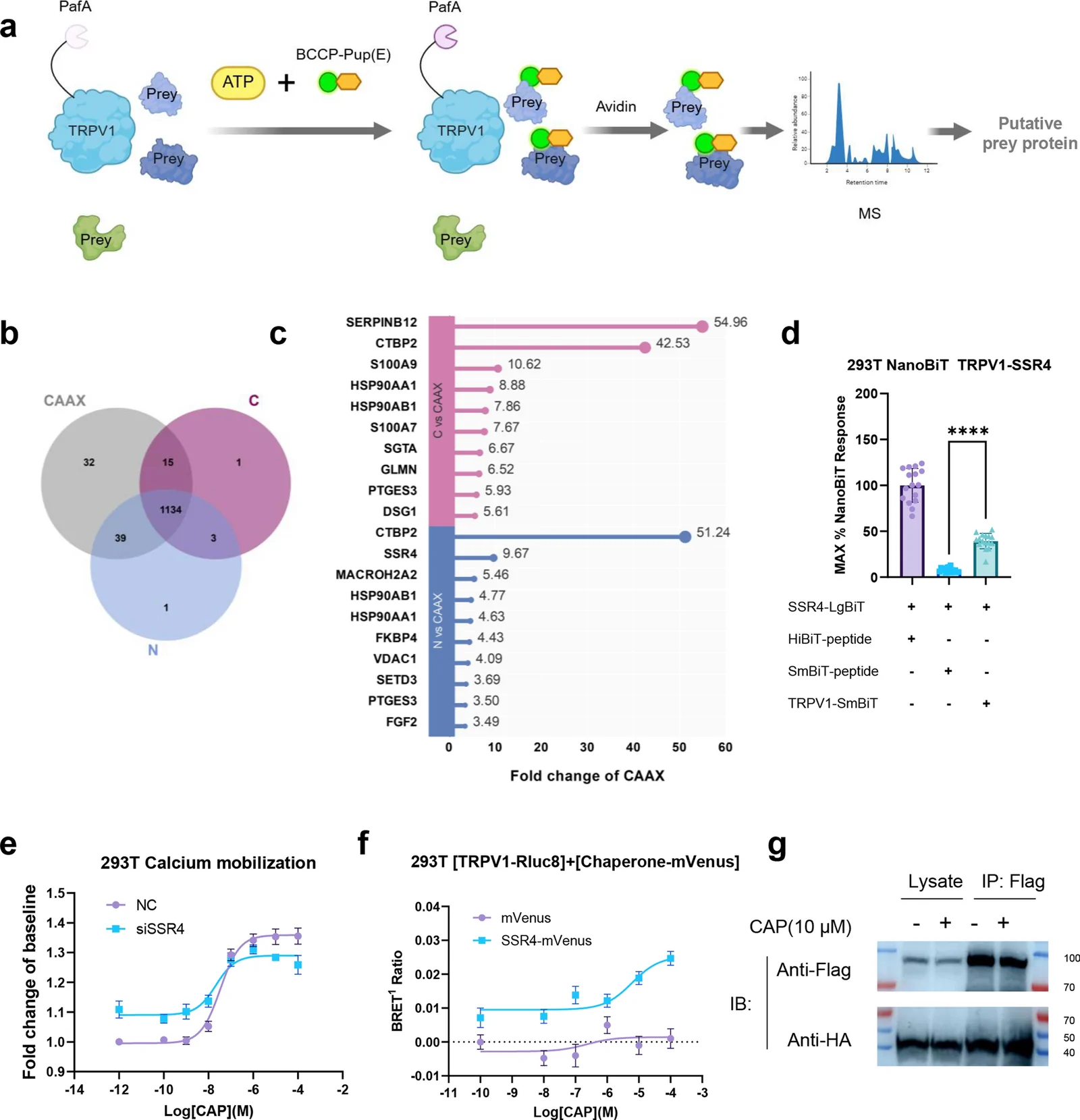
Development of an engineered proximity labeling system to map chaperone interactions with TRPV1. **a** Schematic diagram of the PUP-IT system for identifying potential TRPV1-interacting proteins (created in https://BioRender.com) in 293 T cells. **b** Identification of TRPV1 molecular chaperones using PUP-IT-mass spectrometry. Identification of protein species in the PafA-Flag-CAAX control group, TRPV1-PafA-Flag proximity labeling group, and PafA-TRPV1-Flag proximity labeling group by mass spectrometry. CAAX, C-terminal CaaX prenylation motif used to target PafA to the plasma membrane; N, N-terminal PafA fusion (PafA–TRPV1–Flag); C, C-terminal PafA fusion (TRPV1–PafA–Flag). PafA–Flag–CAAX served as the membrane-associated background control. **c** Differential proteins in the TRPV1-PafA-Flag (C) and PafA-TRPV1-Flag (N) proximity labeling groups compared to the control group (CAAX). **d** Interactions of SSR4 with TRPV1 detected by NanoBiT (*n* = 9–16 independent biological replicates) in 293 T. **e** Effect of SSR4 on TRPV1 calcium mobilization. Fluo4-AM assay to detect the impact of candidate protein knockdown on intracellular calcium levels (*n* = 3 independent biological replicates) in 293 T. **f** Based on BRET technology, the regulatory effect of capsaicin on the TRPV1–SSR4 interaction was determined by detecting its effects on the BRET signal responses between TRPV1-Rluc8 and mVenus, as well as between TRPV1-Rluc8 and SSR4-mVenus (*n* = 3 independent biological replicates) in 293 T. **g** CO-IP experiment verifying the expression levels of Flag and HA after capsaicin treatment (*n* = 3 independent biological replicates) in 293 T. Data represent mean ± SD. *P < 0.05, **P < 0.01, ***P < 0.001, ****P < 0.0001, ns: not significant. *P* values were obtained with one-way ANOVA followed by Tukey’s multiple comparisons tests. CAP, capsaicin

The observation that blockade of canonical TRPV1 channel gating failed to eliminate the anti-inflammatory effect of capsaicin prompted us to search for TRPV1-interacting proteins that might mediate this response. Building on our previous identification of a TRPV1–SARM1 anti-inflammatory axis [19], we established two proximity-labeling systems, PUP-IT and TurboID, to biotinylate proteins in the vicinity of TRPV1 for mass spectrometry-based screening (Fig. 3a, Figs. S9 and S10).

For PUP-IT, PafA was fused to either the N or the C terminus of TRPV1, and a membrane-localized PafA-CAAX construct was used as a control [32]. Immunofluorescence and Western blot analyses confirmed specific biotinylation in the PafA-fused groups (Fig. S9a, b). Comparable labeling was also observed with the TurboID system (Fig. S10a–e). Because PUP-IT showed better labeling performance under our experimental conditions, it was selected for subsequent proteomic analysis (Fig. S10f, g). Streptavidin pull-down followed by mass spectrometry identified 1220 proteins in the PafA-Flag-CAAX control group, 1153 proteins in the TRPV1-PafA-Flag group, and 1177 proteins in the PafA-TRPV1-Flag group (Fig. 3b, c). Candidate TRPV1-interacting proteins were prioritized based on three criteria: enrichment over the control group (ratio > 3), detection in both N-terminal and C-terminal labeling groups, and potential relevance to inflammatory signaling. This analysis yielded eight candidates: CTBP2, SSR4, VDAC1, HSP90AA1, FKBP4, SERPINB12, S100A9, and PTGES3 (Fig. 3c).

To further evaluate their functional relationship with TRPV1, we performed knockdown of each candidate (Fig. S11) and assessed capsaicin-induced calcium mobilization (Fig. 3e, Fig. S12a) to assess whether the candidate proteins may influence the functional state of TRPV1. Comparison of EC_50_, span, and bottom values (Table S5) showed that all candidates affected the TRPV1-mediated calcium responses to varying degrees, supporting a functional association with TRPV1. Based on these results, SSR4, CTBP2, S100A9, HSP90AA1, and SERPINB12 were selected for interaction testing using the NanoBiT assay (Fig. S12b). Among them, SSR4, S100A9, and HSP90AA1 generated robust luminescence signals when co-expressed with TRPV1, whereas SERPINB12 and CTBP2 did not (Fig. 3d, Fig. S12c–f), indicating that the former three proteins are likely to physically associate with TRPV1.

We next asked whether any of these interactions are selectively regulated by capsaicin. BRET assays showed that capsaicin, dihydrocapsaicin, and their mixture significantly enhanced the interaction between TRPV1 and SSR4, whereas no comparable change was observed for the other candidate pairs (Fig. 3f, Fig. S13a–j, Fig. S14a–l). Co-immunoprecipitation further confirmed this interaction: HA-tagged SSR4 was detected in Flag-TRPV1 pull-downs, and capsaicin increased the amount of SSR4 co-precipitated with TRPV1 without altering the total input level (Fig. 3g; Fig. S13k, l). Together, these findings identify SSR4 as a capsaicin-responsive, TRPV1-associated protein and suggest it as a candidate mediator of the anti-inflammatory effects of capsaicin.

### TRPV1–SSR4 interaction is crucial for the capsaicin-induced suppression of microglial inflammatory response

**Fig. 4:**
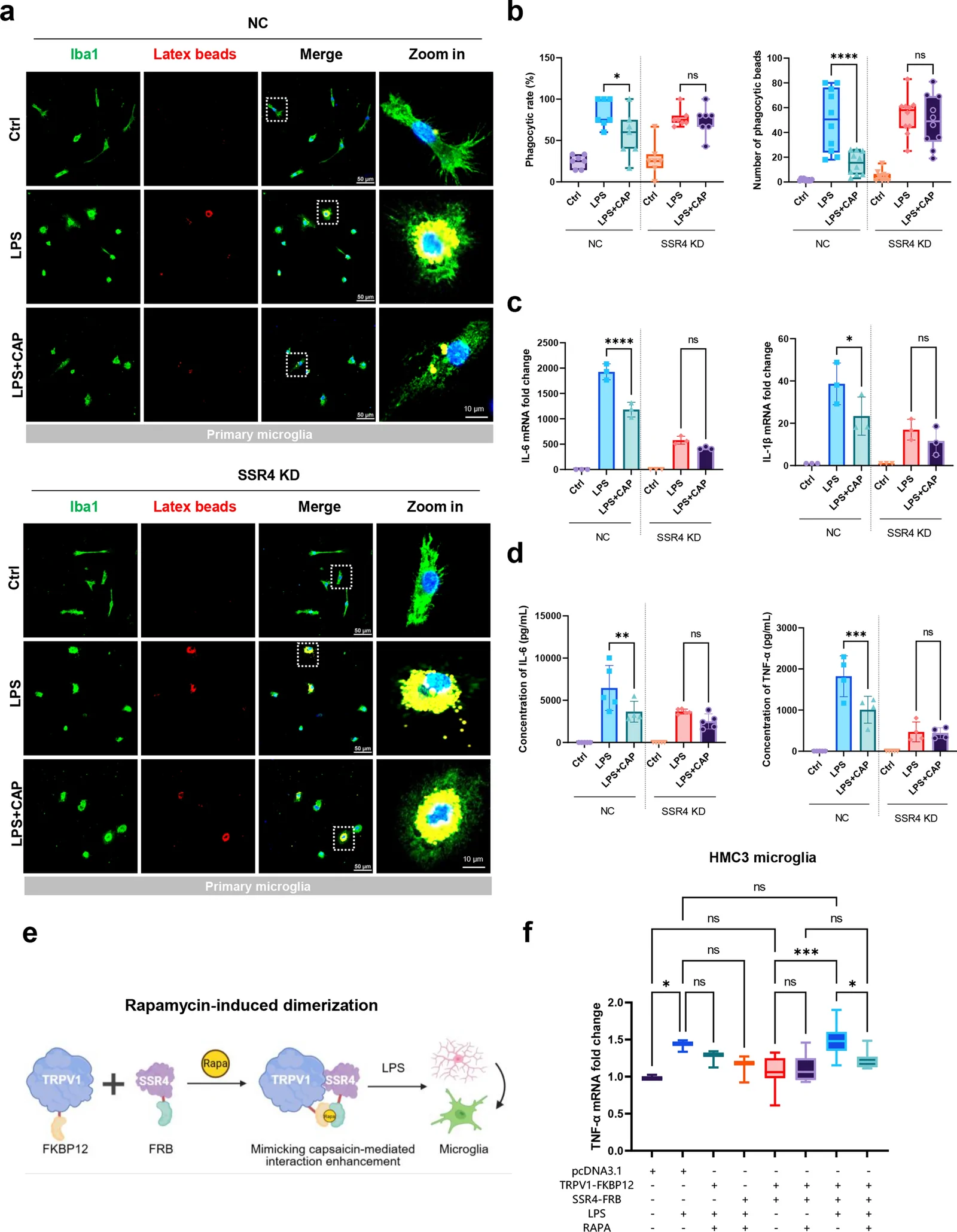
The interaction between TRPV1 and SSR4 underlies the anti-inflammatory effect. **a** Effect of SSR4 on the capsaicin-mediated inhibition of pathological phagocytosis in primary microglia. Imaging of microglia (green) and fluorescent microspheres (red) in the NC group and after SSR4 KD. NC: non-targeting negative-control siRNA. Scale bars, 50 μm in overview images and 10 μm in enlarged images. **b** Statistical analysis of microglial phagocytosis rate (left, each dot represents one individual cell. A total of *n* = 7 cells were analyzed from *n* = 3 independent experiments). Statistical analysis of the average number of microspheres phagocytosed by microglia (right, each dot represents one individual cell. A total of *n* = 10 cells were analyzed from *n* = 3 independent experiments). **c, d** Changes in the regulation of capsaicin on microglial inflammation after SSR4 KD (*n* = 3 independent biological replicates). Regulation of capsaicin on the mRNA (**c**) and protein (**d**) levels of inflammatory factors in LPS-induced microglia after SSR4 KD. **e** Rapamycin-induced dimerization was used to mimic capsaicin-mediated enhancement of the TRPV1–SSR4 interaction and evaluate its effect on LPS-induced microglial responses (created in https://BioRender.com). **f** Quantification of TNF-α mRNA expression in HMC3 microglia after chemically induced TRPV1–SSR4 dimerization under LPS stimulation. Data represent mean ± SD. *P < 0.05, **P < 0.01, ***P < 0.001, ****P < 0.0001, ns: not significant. *P* values were obtained with one-way ANOVA followed by Tukey’s multiple comparisons tests. CAP, capsaicin

Having established that capsaicin selectively enhances TRPV1–SSR4 interaction, we next asked whether SSR4 is necessary for the anti-inflammatory effect of capsaicin in microglia. To address this, SSR4 was knocked down (Fig. S11b, c) and microglial phagocytosis was evaluated using fluorescent microspheres. In control cells, capsaicin significantly reduced the phagocytic rate and decreased the average number of engulfed microspheres per cell, indicating suppression of inflammation-associated phagocytic activation (Fig. 4a, b). In contrast, after SSR4 knockdown, capsaicin no longer significantly reduced either parameter compared with the LPS group (Fig. 4a, b). Thus, the inhibitory effect of capsaicin on pathological microglial phagocytosis depends on the presence of SSR4.

We next examined inflammatory cytokine expression under the same conditions. In control BV2 microglia, capsaicin significantly reduced IL-6 and IL-1β expression, whereas SSR4 knockdown markedly weakened or abolished this suppression (Fig. 4c, d). To verify the specificity of the SSR4 knockdown phenotype, we tested two independent siRNAs. Following knockdown with either SSR4 siRNA, capsaicin failed to induce any further reduction in the levels of these two cytokines (Fig. S11d). Thus, the two independent SSR4 siRNAs yielded consistent knockdown effects and functional phenotypes. These results indicate that SSR4 is required not only for the effect of capsaicin on phagocytosis, but also for its broader inhibition of microglial inflammatory activation.

Because SSR4 depletion also reduced IL-6 expression in LPS-stimulated cells in the absence of capsaicin, we next compared this effect with that of TRPV1 depletion. In BV2 cells, SSR4 knockdown reduced the LPS-induced IL-6 mRNA expression relative to the siNC group, whereas TRPV1 knockdown produced a comparable reduction (Fig. S15a). Combined knockdown of SSR4 and TRPV1 did not further decrease IL-6 expression compared with either alone. In addition, capsaicin reduced IL-6 expression in control cells but produced no detectable further reduction following SSR4 knockdown, TRPV1 knockdown, or combined knockdown (Fig. S15a). If SSR4 and TRPV1 regulated IL-6 expression through fully independent mechanisms, their combined depletion might be expected to produce a greater inhibitory effect. Therefore, the observed non-additive effects are consistent with SSR4 and TRPV1 functioning within the same pathway or converging on a common regulatory mechanism. However, this experiment alone does not establish a direct functional relationship or define the upstream–downstream order between SSR4 and TRPV1.

To gain insights into the downstream pathways associated with this axis, we performed transcriptomic analysis of capsaicin-treated microglia (Fig. S15b–d). DEGs were enriched in processes related to transport, signal transduction, and immune regulation; in cellular components associated with membranes; and in molecular functions related to protein binding. KEGG analysis further highlighted Toll-like receptor signaling, NOD-like receptor signaling, and apoptosis-related pathways (Fig. S15d), suggesting that the TRPV1–SSR4 axis may coordinate the ER-associated protein handling with the inflammatory signaling in microglia.

Finally, to test whether the enhanced TRPV1–SSR4 association is itself sufficient to dampen inflammation, we established a rapamycin-inducible heterodimerization system in which TRPV1 was fused to FKBP12 and SSR4 was fused to FRB (Fig. 4e). In LPS-stimulated microglia, rapamycin-induced TRPV1–SSR4 dimerization significantly reduced TNF-α mRNA expression, whereas rapamycin treatment in the corresponding control group did not produce a comparable effect (Fig. 4f). Together, these data indicate that capsaicin suppresses microglial inflammatory activation by promoting the TRPV1–SSR4 interaction.

### The transmembrane region of TRPV1 mediates SSR4 recruitment

**Fig. 5:**
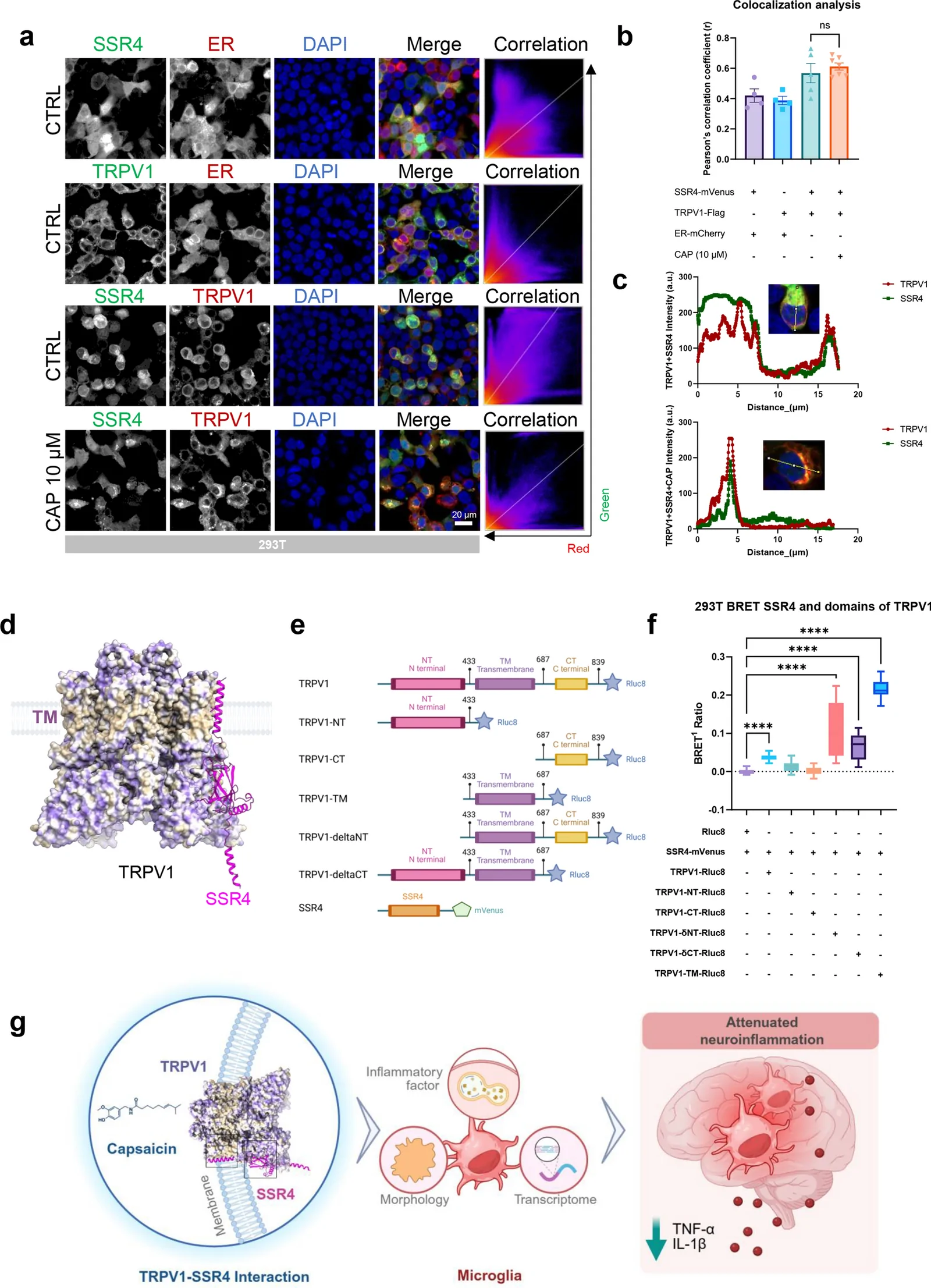
The TRPV1 transmembrane region mediates SSR4 recruitment. **a–c** Representative immunofluorescence images show pairwise colocalization of SSR4 with the ER marker, TRPV1 with the ER marker, and SSR4 with TRPV1 under control or capsaicin-treated conditions. Individual channels are shown in grayscale, and merged images are shown in pseudocolor. Scatter plots and corresponding quantitative analyses show the degree of colocalization. Scale bars, 20 μm. **d** AlphaFold3-predicted interaction residues between TRPV1 and SSR4. **e**, **f** Exploration of TRPV1 domains interacting with SSR4 using BRET technology. Truncated regions of TRPV1: full-length TRPV1, N-terminal region, C-terminal region, transmembrane segment, N-terminal truncated region, and C-terminal truncated region (**e**). BRET response values of SSR4 interacting with the TRPV1 domains (**f**) (*n* = 3 independent biological replicates).** g** Capsaicin enhances the TRPV1–SSR4 interaction in microglia, thereby modulating microglial morphology, inflammatory factor expression, and transcriptomic responses, ultimately attenuating neuroinflammation (created in https://BioRender.com). Data represent mean ± SD. ****P < 0.0001, ns: not significant. *P* values were obtained with one-way ANOVA followed by Tukey’s multiple comparisons tests. CAP, capsaicin

To reassess the subcellular distributions of TRPV1 and SSR4, we performed immunofluorescence imaging in fixed and permeabilized HEK293T cells. Partial colocalization was observed between SSR4 and the ER marker, TRPV1 and the ER marker, and TRPV1 and SSR4 (Fig. 5a, b). Capsaicin treatment did not significantly alter the overall TRPV1–SSR4 colocalization. However, the overlapping signals exhibited more punctate following capsaicin treatment (Fig. 5c), indicating local reorganization of the two proteins. Together with the BRET and co-immunoprecipitation data, these findings support the conclusion that capsaicin enhances TRPV1–SSR4 association, although the subcellular site at which this association occurs remains unresolved.

To explore how TRPV1 associates with SSR4 in cells, we first performed AlphaFold3-based structural modeling of the TRPV1–SSR4 complex. The predicted model suggested that SSR4 primarily engages the interface between two neighboring subunits of the TRPV1 tetramer, with the transmembrane region of TRPV1 forming a major putative contact surface (Fig. 5d). To experimentally map the TRPV1 region required for SSR4 recruitment, we generated six Rluc8-tagged TRPV1 constructs, including full-length TRPV1, isolated N-terminal and C-terminal fragments, the transmembrane region, N-terminally truncated as well as C-terminally truncated forms (ΔNT and ΔCT), each transfected together with SSR4-mVenus for BRET analysis (Fig. 5e). When co-expressed in HEK293T cells, the full-length TRPV1, ΔNT, ΔCT, and the transmembrane region produced BRET signals of 0.037 ± 0.008, 0.108 ± 0.064, 0.067 ± 0.032, and 0.219 ± 0.023, respectively (Fig. 5f). Notably, all TRPV1 constructs containing the transmembrane segment retained proximity to SSR4, whereas the isolated transmembrane region construct generated the strongest BRET signal. These results indicate that the transmembrane region of TRPV1 is a major determinant for SSR4 recruitment.

Together, our proximity-labeling proteomic screen, interaction assays, and functional validation support a model in which TRPV1 associates with SSR4 and capsaicin enhances this association, thereby contributing to the suppression of microglial inflammatory responses (Fig. 5g).

## Discussion

In this study, we identified a ligand-regulated TRPV1–SSR4 axis that contributed to the suppression of microglial inflammatory activation in an LPS-induced neuroinflammatory model. Capsaicin reduced inflammatory cytokine production, neuronal apoptosis, microgliosis and microglial morphological activation, and these protective effects were markedly attenuated in TRPV1-deficient mice and TRPV1-depleted microglia (Fig. 1, Figs. S1–S7). However, capsazepine failed to abolish the anti-inflammatory effect of capsaicin, suggesting that this response is not fully attributed to the capsaicin -sensitive canonical channel gating function of TRPV1 (Fig. 2, Fig. S8). Proximity-labeling proteomics subsequently identified SSR4, the TRAPδ subunit of the ER-associated TRAP complex, as a candidate TRPV1-interacting protein. NanoBiT, BRET and co-immunoprecipitation assays provided complementary evidence for the TRPV1–SSR4 association, showing that this association was enhanced by capsaicin (Fig. 3, Figs. S9–S14). Functionally, SSR4 depletion reduced the LPS-induced inflammatory cytokine expression and prevented further suppression by capsaicin. Conversely, chemically induced proximity between TRPV1 and SSR4 partially reproduced the suppression of LPS-induced TNF-α expression. Transcriptomic profiling further linked this axis to inflammation-related pathways (Fig. 4, Fig. S15). Immunofluorescence colocalization analysis revealed colocalization of TRPV1 and SSR4; furthermore, through AI prediction and BRET assays, we preliminarily identified the transmembrane domain of TRPV1 as the likely key domain for binding to SSR4 (Fig. 5). Collectively, these findings support a model in which capsaicin promotes a functional association between TRPV1 and SSR4 that contributes to the suppression of microglial inflammation, although the precise subcellular site and the downstream signaling intermediates remain to be determined (Fig. 5g).

Although primary mouse microglia represent the most physiologically relevant in vitro model, their exclusive use throughout the full mechanistic workflow was not practical. Unlike immortalized cell lines, primary microglia, particularly those obtained from TRPV1-knockout mice, required colony breeding and genotyping, neonatal brain collection, establishment of mixed-glial cultures, and subsequent microglial isolation. Moreover, each preparation yielded a limited number of cells, which constrained experiments involving multiple treatment groups, genetic manipulation, biochemical assays, and omics-level analyses. Therefore, primary microglia and in vivo models were used to establish the key inflammatory phenotypes, whereas BV2 and HMC3 cells served as complementary systems for mechanistic and biochemical analyses; the consistent qPCR responses observed in primary microglia and BV2 cells further support the relevance of the cell-line findings.

LPS-induced microglial activation remained intact in TRPV1-deficient mice (Fig. 1d, e and Fig. 2a, b), suggesting that TRPV1 is not essential for initiating LPS-induced activation, which is primarily mediated by TLR4-dependent signaling. Nevertheless, brain IL-6 and TNF-α levels after LPS challenge were lower in *Trpv1* KO than in WT mice (Fig. 1d, e), suggesting that endogenous TRPV1 contributes to the inflammatory responsiveness to LPS rather than to its initiation. We therefore interpret TRPV1 as a modulatory receptor/channel that shapes the downstream inflammatory output of LPS-activated microglia, potentially through previously reported calcium-dependent mechanisms and, as shown in this study, through the TRPV1–SSR4 axis.

Importantly, the inflammatory phenotype caused by TRPV1 deletion should be distinguished from the response induced by pharmacological activation of TRPV1. Global TRPV1 deletion reports the consequences of constitutive TRPV1 loss, which may include compensatory and cell-nonautonomous changes, whereas capsaicin treatment reports ligand-evoked response of the intact receptor. Consistent with this, capsaicin lost its anti-inflammatory efficacy in the *Trpv1* KO background, demonstrating that TRPV1 is required for capsaicin-mediated protection. Together, these results support a context-dependent role of TRPV1 in microglial inflammatory regulation, rather than a linear model in which TRPV1 is exclusively pro- or anti-inflammatory.

To investigate the mechanism underlying this noncanonical effect, we combined proximity labeling, proteomic screening, interaction assays, and functional validation. This approach identified SSR4 as a capsaicin-responsive TRPV1-associated protein. SSR4 was first nominated through proximity-labeling-based screening and was subsequently validated by calcium-response assays, NanoBiT, BRET, and co-immunoprecipitation. Among the candidate interactors examined, SSR4 was distinguished by its selective enhancement of interaction with TRPV1 upon stimulation with capsaicin and related capsaicinoids. Thus, SSR4 appears not only to reside in proximity to TRPV1, but also to function as a regulated interaction partner whose association with TRPV1 is promoted by ligand engagement.

Functionally, SSR4 was required for the anti-inflammatory action of capsaicin in microglia. SSR4 knockdown weakened or abolished the ability of capsaicin to suppress inflammatory cytokine expression and inflammation-associated phagocytic activation. Conversely, forced interaction of TRPV1 and SSR4 using a rapamycin-inducible heterodimerization system reduced TNF-α expression in LPS-stimulated microglia. These complementary loss- and gain-of-function results support a causal role for TRPV1–SSR4 interaction in limiting microglial inflammatory activation. Therefore, the TRPV1–SSR4 axis represents a mechanism by which ligand-bound TRPV1 can regulate inflammatory output through protein interaction in addition to canonical ion-conduction mechanisms.

The identity of SSR4 provides a potential link between TRPV1 signaling and ER-associated regulation. SSR4, also known as TRAPδ, is a component of the ER translocon-associated protein complex involved in the handling of nascent secretory and membrane proteins [33, 34]. Given that microglial activation requires coordinated regulation of membrane trafficking, cytokine production, and innate immune signaling, TRPV1–SSR4 coupling may connect membrane receptor activation to intracellular protein-processing and inflammatory regulatory networks. Consistent with this possibility, transcriptomic analysis of capsaicin-treated microglia highlighted pathways related to transport, immune regulation, Toll-like receptor signaling, NOD-like receptor signaling, and apoptosis. Although SSR4 is best characterized as the TRAPδ subunit of the ER-associated TRAP complex, our findings raise the possibility that it also participates in a context-dependent association with TRPV1. However, the direct signaling intermediates remain unknown, and pathway enrichment does not establish direct regulation of these pathways by SSR4 or the TRPV1–SSR4 complex.

Our structural analyses provide an initial framework for understanding how TRPV1 recruits SSR4. AlphaFold3-based modeling suggested that SSR4 interacts with an interface between neighboring TRPV1 subunits, whereas BRET-based domain mapping showed that the transmembrane region of TRPV1 is sufficient for SSR4 association. The strong interaction signal produced by the isolated transmembrane region suggests that this region contains the core determinant for SSR4 recruitment. These findings raise the possibility that ligand-induced conformational changes within the membrane-embedded region of TRPV1 may promote SSR4 coupling and thereby bias TRPV1 toward a noncanonical anti-inflammatory signaling state. Further structural and live-cell imaging studies will be required to directly test this model.

Several limitations should be considered. First, the in vivo experiments were performed in an acute LPS-induced neuroinflammatory model, and whether the TRPV1–SSR4 axis operates similarly in chronic neurodegenerative diseases remains unknown. Second, although we establish a regulated interaction, functional requirement, and structural plausibility, the downstream signaling that links TRPV1–SSR4 coupling to reduced inflammatory gene expression remains to be fully resolved. Third, neurodegenerative diseases involve complex crosstalk among microglia, astrocytes, neurons, endothelial cells, and peripheral immune cells [35–37], that we did not comprehensively address in this study. Future work should examine the TRPV1–SSR4 axis in chronic disease models, define its spatiotemporal dynamics in microglia, and determine whether the interaction can be selectively modulated without broadly activating canonical TRPV1 channel gating.

A further limitation concerns the transcriptomic analysis, for which RNA-seq was performed with a single validated TRPV1 siRNA rather than multiple independent siRNA constructs or primary microglia from *Trpv1* KO mice. To limit siRNA-specific effects, we validated three independent TRPV1 siRNAs by RT-qPCR and western blotting, selected one representative siRNA with comparable knockdown efficiency for RNA-seq, and confirmed selected DEGs by RT-qPCR. We therefore interpret the RNA-seq dataset as pathway-level evidence for TRPV1-associated inflammatory regulation, rather than as proof that each individual differentially expressed gene is directly regulated by TRPV1.

Finally, the in vivo dosing schedule was chosen in light of the rapid brain entry of capsaicin after peripheral administration. A recent simultaneous blood–brain microdialysis study in rats [38] reported that intraperitoneal capsaicin at 2 mg/kg produced a striatal extracellular *C*_max_ of 52.65 ng/mL (~ 170 nM), with a *T*_max_ of 30 min, supporting brain entry within the pretreatment window used in our study. Although our capsaicin dose was lower than that used in the published pharmacokinetics study, the anti-inflammatory effect of capsaicin was abolished by TRPV1 deficiency, indicating functional target engagement in our model. We did not directly measure brain exposure, unbound concentration, or protein binding of capsazepine after systemic administration. Therefore, the in vivo capsazepine experiment should be read as supportive pharmacological evidence, whereas the *Trpv1* KO experiments provide the primary evidence for TRPV1 dependence. Future pharmacokinetics/pharmacodynamics studies should define the brain *C*_max_,* t*_1/2_, unbound exposure, and receptor occupancy of both capsaicin and capsazepine in the LPS-induced neuroinflammation model.

In summary, our findings reveal a novel mechanism by which capsaicin suppresses microglial inflammatory activation through TRPV1–SSR4 interaction. This work expands the functional framework of TRPV1 beyond ion conduction, identifies SSR4 as a key mediator of TRPV1-dependent anti-inflammatory signaling in microglia, and highlights the translational potential of natural TRPV1 agonists and the TRPV1–SSR4 interoceptive signaling for treating neuroinflammation-associated neurodegeneration.

## Supplementary Information

Additional file 1: **Fig. S1**. TRPV1 receptor expression in microglia populations. **Fig. S2**. TRPV1 deficiency modulates microglial transcriptomic profiles. **Fig. S3**. TRPV1 deficiency modulates glial inflammatory factor secretion. **Fig. S4**. Effects of TRPV1 deficiency on primary microglial morphology in vitro. **Fig. S5**. Effects of TRPV1 deficiency on microglial morphology in vivo. **Fig. S6**. Capsaicin exerts anti-neuroinflammatory effects through TRPV1 in murine models. **Fig. S7**. The TRPV1 receptor is crucial for the anti-neuroinflammatory effects of capsaicin in vivo. **Fig. S8**. Blocking the channel function of TRPV1 cannot reverse the anti-neuroinflammatory effects of capsaicin. **Fig. S9**. Development of an engineered PUP-IT system to map chaperone interactions with TRPV1. **Fig. S10**. A proximity-labeling system for systematic identification of TRPV1-associated chaperones. **Fig. S11**. KD efficiency of chaperones. **Fig. S12**. TRPV1-chaperone interactions in live cells were validated. **Fig. S13**. Capsaicin facilitates direct binding of TRPV1 to SSR4. **Fig. S14**. Capsaicin cannot promote the binding between TRPV1 and other chaperones. **Fig. S15**. Transcriptomic remodeling of BV2 microglial inflammatory responses following capsaicin treatment. **Table S1**. Key resources. **Table S2**. Sequences of siRNAs used in the study. **Table S3**. Sequences of primers used for quantitative real-time PCR. **Table S5**. Effect of candidate protein knockdown on intracellular calcium levels

Additional file 2: **Table S4**. Information of constructs used in this study

Additional file 3: The full uncropped Gels and Blots images

## Data Availability

All data needed to evaluate the conclusions in the paper are present in the paper and/or the Supplementary Materials. MS generated in this study has been deposited in the ProteomeXchange Consortium (https://proteomecentral.proteomexchange.org) via the iProX partner repository with the dataset identifier PXD066698. Data are accessible via the provided link: https://proteomecentral.proteomexchange.org/cgi/GetDataset?ID=PXD066698 and iProX: https://www.iprox.cn/page/project.html?id=IPX0012747000. The raw data of RNA-seq reported in this paper have been deposited in the Sequence Read Archive (SRA) with BioProject accession PRJNA1295138 and submission ID SUB15639787/SUB15644420. The data are publicly available, accessible via the following links: https://www.ncbi.nlm.nih.gov/sra/PRJNA1295138.

## Abbreviations

TRPV1: Transient receptor potential vanilloid 1
SSR4: Signal sequence receptor subunit 4
SARM1: Sterile alpha and TIR motif-containing 1
PUP-IT: Pupylation-based interaction tagging
BRET: Bioluminescence resonance energy transfer
NanoBiT: NanoLuc binary technology
NC: Negative-control siRNA
ER: Endoplasmic reticulum
