# Supplementary material for: Capsaicin suppresses microglial inflammation through TRPV1–SSR4 interaction
Source: Transl Neurodegener. 2026 Oct 21;15:57. doi: 10.1186/s40035-026-00589-7 (PMC13685340; doi:10.1186/s40035-026-00589-7)
Supplement: Supplementary file 1 [file 40035_2026_589_MOESM1_ESM.docx]

# **Supplementary figures and figure legends**

**Fig. S1 TRPV1 receptor expression in microglia populations.**

**a** Fluorescence co-expression of TRPV1 (red) and Iba1 (green). Scale bars, 50 μm. **b** The percentage of TRPV1-positive microglia was calculated as the number of TRPV1-positive Iba1-positive cells divided by the total number of Iba1-positive cells. **c-d** The knockdown efficiency of three TRPV1 siRNAs was assessed by western blotting. **e** The knockdown efficiency of three TRPV1 siRNAs was assessed by qPCR. NC: non-targeting negative-control siRNA.

**Fig. S2 TRPV1 deficiency modulates microglial transcriptomic profiles.**

**a-c** Changes in transcriptional profiles (**a**), KEGG pathway enrichment (**b**), and GO term enrichment (**c**) in BV2 cells following TRPV1 knockdown under control conditions. **d, f** Changes in anti-inflammatory (**d**) and pro-inflammatory genes (**f**) in BV2 cells following TRPV1 knockdown under LPS stimulation. **e** Changes in GO enrichment in BV2 cells following TRPV1 knockdown under LPS stimulation.

**Fig. S3 TRPV1 deficiency modulates glial inflammatory factor secretion.**

**a-c** The impact of TRPV1 KD on IL-6 (**a**), TNF-α (**b**), and IL-1β (**c**) levels in BV2. **d-f** The impact of TRPV1 KO on IL-6 (**d**), TNF-α (**e**), and IL-1β (**f**) levels in primary microglia. (*n* = 3 independent biological replicates). Data represent mean ± SD, *P* < 0.05, ***P* < 0.01, ****P* < 0.001, *****P* < 0.0001, ns: not significant. *P* values were obtained with one-way ANOVA followed by Tukey’s multiple comparisons tests.

**Fig. S4 Effects of TRPV1 deficiency on primary microglial morphology in vitro.**

**a** Iba1 immunofluorescence staining, segmentation and skeletonization analysis of primary microglia in WT and TRPV1 KO groups. Scale bars, 40 μm. **b-e** Junction number (**b**), branch number (**c**), intersection number within the range from soma to dendritic terminals (**d**), with the corresponding area under the curve calculated (**e**) (Each dot represents one individual cell. A total of *n* = 13-20 cells were analyzed from *n* = 3 independent biological replicates). **f** TRPV1 protein levels were detected in the brain from the WT and TRPV1 KO mice (*n* = 3 independent biological replicates ). **g** Iba1 immunofluorescence staining and skeletonization analysis of primary microglia in WT and TRPV1 KO groups under inflammatory conditions. Scale bars, 40 μm. **h-k** Junction number (**h**), branch number (**i**), intersection number within the range from soma to dendritic terminals (**j**), with the corresponding area under the curve calculated (**k**) (Each dot represents one individual cell. A total of *n* = 18-20 cells were analyzed from *n* = 3 independent biological replicates). Data represent mean ± SD, ns: not significant. *P* values were obtained with two-sided paired ratio Student’s t-test.

**Fig. S5 Effects of TRPV1 deficiency on microglial morphology in vivo.**

**a** Iba1 immunofluorescence staining and sholl analysis of microglia in WT and TRPV1 KO mice. Scale bars, 100 μm and 50 μm. **b-e** Sholl analysis of primary microglia junction number (**b**), branch number (**c**), and intersection number within the range from soma to dendritic terminals (**d**), with the corresponding area under the curve calculated (**e**) (Each dot represents one individual cell. A total of *n* = 8-10 cells were analyzed from *n* = 3 independent biological replicates). **f** Iba1 immunofluorescence staining and sholl analysis of primary microglia in WT and TRPV1 KO mice under inflammatory conditions. Scale bars, 100 μm and 50 μm. **g-j** Sholl analysis of microglia junction number (**g**), branch number (**h**), and intersection number within the range from soma to dendritic terminals (**i**), with the corresponding area under the curve calculated (**j**) (Each dot represents one individual cell. A total of *n* = 8-10 cells were analyzed from *n* = 3 independent biological replicates). Data represent mean ± SD, ns: not significant. *P* values were obtained with two-sided paired ratio Student’s t-test.

**Fig. S6 Capsaicin exerts anti-neuroinflammatory effects through TRPV1 in murine models.**

**a** Immunohistochemical staining of cl-caspase-3 in brain tissues of mice from the WT group, LPS group, LPS + CAP group. Scale bars, 50 μm**. b** Immunohistochemical staining of cl-caspase-3 in brain tissues of mice from the Ctrl group, LPS group, and LPS+CAP group after TRPV1 KO. Scale bars, 50 μm.

**Fig. S7 The TRPV1 receptor is crucial for the anti-neuroinflammatory effects of capsaicin in vivo.**

**a** Iba1 immunofluorescence staining of WT mouse brain tissue sections. **b** Iba1 immunofluorescence staining of mouse brain tissue sections after TRPV1 KO. Scale bars, 100 μm in overview images and 50 μm in enlarged images.

**Fig. S8 Blocking the channel function of TRPV1 cannot reverse the anti-neuroinflammatory effects of capsaicin.**

**a** Immunofluorescence staining of microglial Iba1. Scale bars, 100 μm in overview images and 50 μm in enlarged images. **b** BV2 cells transfected with control siRNA or TRPV1 siRNA were treated with LPS, CAP, capsazepine (CZP), or CAP plus CZP as indicated. IL-6 mRNA expression was measured by RT-qPCR.

**Fig. S9 Development of an engineered PUP-IT system to map chaperone interactions with TRPV1.**

**a** Fluorescence expression of Flag and biotin in HEK-293T cells after transfection with PafA proximity labeling-related plasmids. TRPV1–PafA denotes the construct in which PafA was fused to the C terminus of TRPV1, whereas PafA–TRPV1 denotes the construct in which PafA was fused to the N terminus of TRPV1. Scale bars, 50 μm. **b** Pull-down assay verification of PafA proximity labeling system activity (*n* = 3 independent biological replicates). Western blotting analysis of Flag and biotin-labeled protein expression in different groups (upper). Flag and biotin-labeled protein expression in different groups after pull-down with streptavidin magnetic beads (lower).

**Fig. S10 A proximity-labeling system for systematic identification of TRPV1-associated chaperones.**

**a** Schematic diagram of the TurboID system for identifying potential TRPV1-interacting proteins (created in https://BioRender.com). **b-c** Verification of the plasmid activity in the proximity labeling system (*n* = 3 independent biological replicates). Changes in intracellular calcium levels after treatment with gradient concentrations of capsaicin and a fixed concentration of capsazepine (**b**). Changes in intracellular calcium levels after treatment with a fixed concentration of capsaicin and gradient concentrations of capsazepine (**c**) in 293T. **d** Immunofluorescence verification of TurboID proximity labeling system activity. Fluorescence expression of Flag and biotin in HEK-293T cells after transfection with TurboID proximity labeling-related plasmids (*n* = 3 independent biological replicates). Scale bars, 50 μm. **e** Pull-down assay verification of TurboID proximity labeling system activity (*n* = 3 independent biological replicates). Western blotting analysis of Flag and biotin-labeled protein expression in different groups (left). Flag and biotin-labeled protein expression in different groups after pull-down with streptavidin magnetic beads (right). **f-g** Comparison of the PUP-IT and TurboID proximity labeling systems (*n* = 3 independent biological replicates). Silver staining to detect biotinylated proteins in each group (**f**). Western blotting to detect biotinylated proteins in each group (**g**).

**Fig. S11 KD efficiency of chaperones.**

**a** Design siRNA targeting CTBP2, HSP90AA1, SERPINB12, S100A9, SSR4 (siSSR4-1, siSSR4-2), FKBP4, PTGES3, and VDAC1; transfect siRNA into CHO-K1 cells and assess the KD efficiency. **b-c** Western blot analysis of the knockdown efficiency of two SSR4 siRNAs. **d** IL-6 and IL-1β mRNA expression following LPS stimulation with or without capsaicin. Data represent three independent experiments. ns, not significant. **P*  <  0.05, ***P*  <  0.01 and **** *P*  < 0.0001. (*n* = 3 independent biological replicates).

**Fig. S12 TRPV1-chaperone interactions in live cells were validated.**

**a** Effect of candidate proteins on TRPV1 calcium mobilization in 293T. Fluo4-AM assay to detect the impact of candidate protein KD on intracellular calcium levels (*n* = 3 independent biological replicates). **b** Exploring the interaction between candidate proteins and TRPV1 based on NanoBiT technology (created in https://BioRender.com). **c-f** Interactions of S100A9 (**c**), HSP90AA1 (**d**), SERPINB12 (**e**), and CTBP2 (**f**) with TRPV1 (*n* = 9-16 independent biological replicates). Data represent mean ± SD. * *P* < 0.05, ***P* < 0.01, ****P* < 0.001, *****P* < 0.0001, ns: not significant. *P* values were obtained with one-way ANOVA followed by Tukey’s multiple comparisons tests.

**Fig. S13 Capsaicin facilitates direct binding of TRPV1 to SSR4.**

**a** Schematic diagram of exploring the regulation of capsaicin on the TRPV1-chaperone axis based on BRET technology (created in https://BioRender.com ) in 293T. **b-j** Exploring the regulation of TRPV1–SSR4 interaction by different ligands based on BRET technology. Capsaicin (**b, e**), Dihydrocapsaicin (DCAP) (**c, f**), Mixture of capsaicin and dihydrocapsaicin (CAO) (**d, g**), capsazepine (capsazepine) (**h**), Piperine (**i**), and Evodiamine (**j**) on the BRET signal response of TRPV1-Rluc8 with mVenus, and TRPV1-Rluc8 with SSR4-mVenus (*n* = 3 independent biological replicates). **k** Schematic diagram illustrating the effect of capsaicin on TRPV1–SSR4 binding based on CO-IP technology (created in https://BioRender.com). **l** CO-IP analysis of the relative levels of Flag- and HA-tagged proteins after capsaicin treatment (*n* = 3 independent biological replicates). Data represent mean ± SD. **P* < 0.05, ***P* < 0.01, ****P* < 0.001, *****P* < 0.0001, ns: not significant. *P* values were obtained with one-way ANOVA followed by Tukey’s multiple comparisons tests.

**Fig. S14 Capsaicin cannot promote the binding between TRPV1 and other chaperones.**

**a-f** Exploring the regulation of TRPV1-HSP90AA1 interaction by different ligands based on BRET technology in 293T. Capsaicin (**a**), DCAP (**b**), CAO (**c**), capsazepine (**d**), Piperine (**e**), and Evodiamine (**f**) on the BRET signal response of TRPV1-Rluc8 and HSP90AA1-mVenus (*n* = 3 independent biological replicates). **g-l** Exploring the regulation of TRPV1-S100A9 interaction by different ligands based on BRET technology. Capsaicin (**g**), DCAP (**h**), CAO (**i**), capsazepine (**j**), Piperine (**k**), and Evodiamine (**l**) on the BRET signal response of TRPV1-Rluc8 with mVenus, and TRPV1-Rluc8 with S100A9-mVenus (*n* = 3 independent biological replicates).

**Fig. S15 Transcriptomic remodeling of BV2 microglial inflammatory responses following capsaicin treatment.**

**a** IL-6 mRNA expression detection after single and combined knockdown of SSR4 and TRPV1 in LPS-stimulated BV2 cells. **b** Volcano plot of differentially expressed genes (DEGs). The plot shows the DEGs between the LPS + CAP group and LPS group. Red dots indicate significantly upregulated genes, blue dots indicate significantly downregulated genes and gray dots represent genes with no significant changes. **c** GO functional enrichment analysis. CAP exerts differential effects on LPS-induced BV2 microglia across Biological Process (BP), Cellular Component (CC), and Molecular Function (MF) categories. **d** KEGG pathway enrichment analysis. Pathways enriched among DEGs in the LPS + CAP vs. LPS comparison. The rich factor represents the ratio of DEGs in a pathway to the total number of genes in that pathway. The dot size corresponds to the number of significantly enriched genes, and the color indicates the *P*-value. CAP, capsaicin.

**Table S1. Key resources**

| **REAGENT or RESOURCE** | **SOURCE** | **IDENTIFIER** |
| --- | --- | --- |
| **Antibodies** |  |  |
| Anti Iba1, Rabbit (for Immunocytochemistry) | FUJIFILM Wako | Cat# 019-19741 |
| Anti-TRPV1 antibody, Rabbit | Abcam | Cat# ab3487 |
| Anti-TRPV1 antibody, Rabbit | Alomone labs | Cat# ACC-030 |
| Anti-TRPV1 antibody, Mouse | Santa Cruz | Cat# sc-398417 |
| Goat anti-Mouse IgG (H+L)  Secondary Antibody, Alexa Fluor™ Plus 488 | Cell Signaling Technology | Cat# 4408S |
| Goat anti-Rabbit IgG (H+L)  Secondary Antibody, Alexa Fluor™ Plus 594 | Cell Signaling Technology | Cat# 8889S |
| Cleaved Caspase-3 (Asp175) Antibody | Cell Signaling Technology | Cat# 9661S |
| Multi-rAb® Polymer HRP-Goat Anti-Rabbit Recombinant Secondary Antibody (H+L) | Proteintech | Cat# RGAR011 |
| CoraLite®594-conjugated DYKDDDDK Monoclonal antibody | Proteintech | Cat# CL594-66008 |
| Anti-DDDDK tag (Binds to FLAG® tag sequence) | Abcam | Cat# ab205606 |
| Anti-HA Tag Rabbit Polyclonal Antibody | Adamas life | Cat# P9125 |
| **Chemicals** |  |  |
| Capsaicin | MedChemExpress LLC | Cat# HY-10448 |
| Capsazepine | MedChemExpress LLC | Cat# HY-15640 |
| DMEM | BasalMedia | Cat# L110KJ |
| DMEM/F12 (1:1) | Gibco | Cat# 11320-033 |
| Opti-MEM | Gibco | Cat# 31985-070 |
| FBS | Ozfan | Cat# FBSKM0502 |
| 1% L-alanyl-L-glutamine solution | BasalMedia | Cat# S240JV |
| Penicillin–Streptomycin 100× | BasalMedia | Cat# S110JV |
| PBS | BasalMedia | Cat# B320KJ |
| 0.25%-Typsin-EDTA | Gibco | Cat# 25200-072 |
| 4% PFA | Titan Scientific Co. Ltd | Cat# F8011 |
| DMSO | Titan Scientific Co. Ltd | Cat# F8011 |
| TritonX-100 | Merck | Cat# T8787 |
| ProLong™ Gold Antifade Mountant | Thermo Fisher Scientific | Cat# P36930 |
| HBSS | BasalMedia | Cat# B410KJ |
| LPS | Merck | Cat# L4391-1MG |
| Serum-free Cell Cryopreservation Medium | Epizyme | Cat# BY002P |
| DNA Marker D (100~2000 bp) | Sangon | Cat# B600335-0250 |
| Agarose, Regular | Sangon | Cat# A620014-0100 |
| 5X TBE Buffer | Sangon | Cat# B548102-0500 |
| 4S Green Plus Nucleic Acid Stain | Sangon | Cat# A616696-0500 |
| Protease inhibitor cocktail | APExBIO | Cat# K1007 |
| Phosphatase inhibitor cocktail | APExBIO | Cat# K1015-A/B |
| PMSF | Beyotime | Cat# ST505 |
| DAPI | Sangon | Cat# E607303-0020 |
| PEG300 | MCE | Cat# HY-Y0873 |
| Tween 80 | MCE | Cat# HY-Y1891 |
| HRP-conjugated Biotin | Sangon | Cat# D111127 |
| Biotin | MCE | Cat# HY-B0511 |
| HRP Streptavidin | Yeasen | Cat# 35103ES60 |
| Streptavidin Beads T3 | APExBIO | Cat# K1301 |
| MonoRab™ Anti-DYKDDDDK Magnetic Beads | Genscript | Cat# L00835-1 |
| Western Rapid Transfer Buffer | Beyotime | Cat# P0575-10L |
| SDS-PAGE Electrophoresis Buffer | Beyotime | Cat# P0014B |
| Fluo 4-AM | DOJINDO | Cat# F311 |
| Probenecid | Sigma-Aldrich | Cat# P8761 |
| Pluronic F-127 | Sigma-Aldrich | Cat# P2443 |
| Furimazine | ChemShuttle | Cat# 185252 |
| Hieff Trans™ Liposomal Transfection Reagent | Yeasen | Cat# 40802ES |
| Coelenterazine h | Yeasen | Cat# 40906ES10 |
| Rapamycin | MedChemExpress LLC | Cat# HY-10219 |
| Aqueous suspension | Merck | Cat# L2778 |
| **Critical commercial assays** |  |  |
| FastPure Cell/Tissue total RNA Isolation Kit V2 | Vazyme | Cat# RC112-01 |
| HiScript Q RT SuperMix for qPCR (+gDNA wiper) | Vazyme | Cat# R323-01 |
| Taq Pro universal SYBR qPCR master mix | Vazyme | Cat# Q712-03 |
| BCA Protein Assay Kit | Beyotime | Cat# P0012 |
| Multicolor prestained protein ladder | Epizyme | Cat# WJ101 |
| Quick Genotyping Assay Kit for Mouse Tail | Beyotime | Cat# D7283M |
| RIPA lysis buffer | Epizyme | Cat# PC102 |
| Mouse TNF-α ELISA Kit | MultiScience | Cat# EK282 |
| Mouse IL-6 ELISA Kit | MultiScience | Cat# EK206 |
| Hematoxylin and Eosin (H&E) Staining Kit | Huntz Jaha | Cat# HJ-H0750-4 |
| Fast Silver Stain Kit | Beyotime | Cat# P0017S |
| CALNP™ RNAi in vitro | D-nano Therapeutics | Cat# DN001-01 |
| **Deposited data** |  |  |
| RNA-seq data of primary astrocytes | This paper | SUB15639787 |
| RNA-seq data of microglia | This paper | SUB15644420 |
| DARTS data of HEK-293T | This paper | PXD066698 |
| **Experimental models: Cell lines/Organisms** |  |  |
| Primary microglia | This paper | N/A |
| BV2 | This paper | N/A |
| Primary astrocytes | This paper | N/A |
| TRPV1^-/-^ primary astrocytes | This paper | N/A |
| HEK-293T | This paper | N/A |
| CHO-K1 | This paper | N/A |
| Mouse: TRPV1^-/-^ | This paper | N/A |
| **Oligonucleotides** |  |  |
| siRNA | This paper | Table S2 |
| Primers for qRT-PCR analysis | This paper | Table S3 |
| **Recombinant DNA** |  |  |
| TRPV1-PafA-Flag | This paper | N/A |
| pc DNA3.1 | This paper | N/A |
| TRPV1-Flag | This paper | N/A |
| BCCP-Pup(E) | This paper | N/A |
| PafA-Flag-CAAX | This paper | N/A |
| TRPV1-TurboID-Flag | This paper | N/A |
| TurboID-TRPV1-Flag | This paper | N/A |
| TRPV1-SmBiT | This paper | N/A |
| SSR4-LgBiT | This paper | N/A |
| S100A9-LgBiT | This paper | N/A |
| HSP90AA1-LgBiT | This paper | N/A |
| SERPINB12-LgBiT | This paper | N/A |
| SSR4-mVenus-HA | This paper | N/A |
| S100A9-mVenus-HA | This paper | N/A |
| HSP90AA1- mVenus-HA | This paper | N/A |
| TRPV1-Rluc8 | This paper | N/A |
| **Software and algorithms** |  |  |
| GraphPad Prism | GraphPad Software | http://www.graphpad.com/ |
| ImageJ | NIH | https://imagej.nih.gov/ij/ |
| BioRender | Website | https://www.biorender.com/ |
| **Instruments** | **Manufacturer** | **Identifier** |
| Ultra-low temperature freezer | Haier | DW-86L626 |
| NanoDrop™ One/OneC Microvolume UV-Vis Spectrophotometer | Thermo Fisher Scientific | ND-ONE-W |
| NanoDrop 2000 UV-Vis spectrophotometer | Thermo Fisher Scientific | ND-2000 |
| Benchtop high-speed refrigerated centrifuge | Eppendorf | 5424 R |
| Biosafety cabinet | Haier | HR1200-IIA2 |
| Two-photon imaging microscope | Nikon | A1R MP+ |
| Axio Imager A2 microscope | Carl Zeiss Microscopy GmbH | Axio Imager A2 |
| PHERAstar FSX multimode microplate reader | BMG LABTECH GmbH | PHERAstar FSX |
| Histological microtome | Leica | Leica RM2235 |
| Digital slide scanner | KFBIO | KFBIO KF-PRO-005 |
| qPCR instrument | Applied Biosystems | A28571 |
| Amersham ImageQuant | Cytiva | ImageQuant 800 |
| Magnetic rack | Cytiva | MagRack 6 |
| HPLC system | Thermo Scientific | UltiMate 3000 |
| Q Exactive HF mass spectrometer | Thermo Scientific | Q Exactive HF |

Table S2. Sequences of siRNAs used in the study.

| **siRNA** | **Sense sequence** | **Antisense sequence** |
| --- | --- | --- |
| CTBP2 | GGUGGAUGAGAAAGCCUUA | UAAGGCUUUCUCAUCCACC |
| SSR4-1/SSR4 | GGAAGGCUCAGAGAAAUAA | UUAUUUCUCUGAGCCUUCC |
| SSR4-2 | ACCUAUGAGGUCAGAUUCU | AGAAUCUGACCUCAUAGGU |
| VDAC1 | UGGAAUUUCAAGCAUAAAU | AUUUAUGCUUGAAAUUCCA |
| HSP90AA1 | GGAUCUACAGGAUGAUCAA | UUGAUCAUCCUGUAGAUCC |
| FKBP4 | GCUCUAUCCCAGCAACAAA | UUUGUUGCUGGGAUAGAGC |
| SERPINB12 | AGUGGGAGAGAGAAUUCAA | UUGAAUUCUCUCUCCCACU |
| S100A9 | GAAAGAGAAGAGAAAUGAA | UUCAUUUCUCUUCUCUUUC |
| PTGES3 | GGCUCAGUGUGGACUUCAA | UUGAAGUCCACACUGAGCC |
| TRPV1-1/TRPV1 | GCGCAUCUUCUACUUCAACUUTT | AAGUUGAAGUAGAAGAUGCGCTT |
| TRPV1-2 | CCAUCCCUCAAGAGUUUGUUUTT | AAACAAACUCUUGAGGGAUGGTT |
| TRPV1-3 | GCCAUGCUCAAUCUGCACAAUTT | AUUGUGCAGAUUGAGCAUGGCTT |

Table S3. Sequences of primers used for quantitative real-time PCR.

| **Primer** | **Forward sequence** | **Reverse sequence** |
| --- | --- | --- |
| TRPV1 | CACCACGGCTGCTTACTATCG | AGAAGAAGTAGACTCCTCCTGACAC |
| IL-6 | TTCTTGGGACTGATGCTGGTGAC | CAGGTCTGTTGGGAGTGGTATCC |
| TNF-α | CGCTCTTCTGTCTACTGAACTTCGG | GTGGTTTGTGAGTGTGAGGGTCTG |
| CTBP2 | GGCAGAGGGTGTGGCAGAG | GGCTAGGCGGCTTAGGACTC |
| SSR4 | GGCGGCGATGGCATCTTTC | TCTGTAGAAATGACGGCATCTGAAG |
| VDAC1 | GTGAATGACGGGACAGAGTTTGG | CGACCTGATACTTGGCTGCTATTC |
| HSP90AA1 | ATCACGAAGCATAACGACGATGAG | TAACCTTTGTTCCACGACCCATTG |
| FKBP4 | GGCGTGCTCAAGGTCATCAAG | TAGCAGCCAGCCAGTGTAGTG |
| SERPINB12 | TTGTGGAGGTGGATGAGATGGG | AGGAAAGGGTGGTTGGCATTAAAC |
| S100A9 | GAAGCACAGTTGGCAACCTTTATG | ATCAGCATCATACACTCCTCAAAGC |
| PTGES3 | GGCTCAGTGTGGACTTCAATAATTG | TCATCATCTGCTCCATCTACTTCTG |
| 18S | AGGGGAGAGCGGGTAAGAGA | GGACAGGACTAGGCGGAACA |

Table S5. Effect of candidate protein knockdown on intracellular calcium levels

| siRNA | Top | Bottom | LogEC_50_ | EC_50_ (M) | Span |
| --- | --- | --- | --- | --- | --- |
| NC | 1.359 | 0.9955 | -7.471 | 3.38×10⁻⁸ | 0.363 |
| siPTGES3 | 1.051 | 0.9961 | -7.664 | 2.17×10⁻⁸ | 0.055 |
| siVDAC1 | 1.359 | 1.08 | -8.068 | 8.55×10⁻⁹ | 0.279 |
| siSSR4 | 1.29 | 1.09 | -7.668 | 2.15×10⁻⁸ | 0.2 |
| siHSP90AA1 | 1.498 | 1.309 | -7.729 | 1.87×10⁻⁸ | 0.19 |
| siFKBP4 | 1.469 | 1.12 | -7.984 | 1.04×10⁻⁸ | 0.35 |
| siS100A9 | 1.055 | 0.8903 | -7.576 | 2.66×10⁻⁸ | 0.165 |
| siCTBP2 | 1.2 | 1.106 | -7.527 | 2.97×10⁻⁸ | 0.095 |
