# Supplementary material for: Capsaicin suppresses microglial inflammation through TRPV1–SSR4 interaction
Source: Transl Neurodegener. 2026 Oct 21;15:57. doi: 10.1186/s40035-026-00589-7 (PMC13685340; doi:10.1186/s40035-026-00589-7)
Supplement: Supplementary file 2 [file 40035_2026_589_MOESM3_ESM.docx]

**Capsaicin suppresses microglial inflammation through TRPV1–SSR4 interaction**

**The full uncropped Gels and Blots images**

**Fig.3g:**

Anti-Flag：

Anti-HA:

**Fig.S1c:**

Anti-TRPV1：

Anti-GAPDH：

**Fig.S4f:**

Anti-TRPV1：

**Fig.S9b:**

Lysate Biotinylated Proteins (SA-HRP):

Lysate Anti-Flag：

After streptavidin pull down Biotinylated Proteins (SA-HRP):

After streptavidin pull down Anti-Flag：

**Fig.S10e:**

Lysate Biotinylated Proteins (SA-HRP):

Lysate Anti-Flag：

After streptavidin pull down Biotinylated Proteins (SA-HRP):

After streptavidin pull down Anti-Flag：

**Fig.S10f:**

Silver staining:

**Fig.S10g:**

SA-HRP:

**Fig.S11b:**

Anti-GAPDH:

Anti-SSR4:
